# Environmental adaptation in wheat landraces: integrating physiological, molecular, and ecological mechanisms for climate-resilient agriculture

**DOI:** 10.3389/fpls.2026.1942405

**Published:** 2026-10-01

**Authors:** Dali Vilma Francis, Malu Kishorkumar, Tarfa Abdulla, Abdul Jaleel, Habiba Alsafar, Ayesha Al Dhaheri, Shyam S. Kurup

**Affiliations:** 1 Department of Integrative Agriculture, College of Agriculture and Veterinary Medicine, United Arab Emirates University, Al Ain, United Arab Emirates; 2 Center of Biotechnology, Khalifa University, Abu Dhabi, United Arab Emirates; 3 Department of Nutrition and Health, College of Medicine and Health Sciences, United Arab Emirates University, Al Ain, United Arab Emirates

**Keywords:** abiotic stress, arid regions, climate-smart agriculture, environmental adaptation, genetic diversity, hormonal regulation, omics technologies, wheat breeding

## Abstract

Climate change poses an increasing threat to wheat production, particularly in arid and semi-arid regions where rising temperatures, water scarcity, soil degradation, and salinity interact to reduce crop productivity and stability. Wheat landraces constitute an invaluable reservoir of adaptive genetic diversity that has evolved through centuries of natural and farmer-mediated selection under resource-limited environments. This review provides a comprehensive synthesis of the mechanisms underlying environmental resilience in wheat landraces, integrating evidence across physiological, biochemical, hormonal, molecular, ecological, and agronomic scales within a single framework. It examines how coordinated adaptive strategies—including stress escape, avoidance, tolerance, and recovery—interact with root system architecture, rhizosphere processes, plant–microbe associations, and resource-use efficiency to enhance resilience under multiple abiotic stresses. Recent advances in genomics, transcriptomics, proteomics, metabolomics, pangenomics, and multi-omics integration have substantially improved our understanding of the genetic and molecular basis of environmental adaptation, creating new opportunities for the identification and deployment of resilience-associated traits. The review further discusses how these discoveries are being translated into climate-smart wheat improvement through genome-wide association studies, marker-assisted and genomic selection, speed breeding, gene editing, and artificial intelligence-assisted phenotyping. In addition, sustainable agronomic management, conservation strategies, farmer participation, and policy frameworks are considered as complementary components supporting the effective utilization of wheat landraces. By integrating adaptive biology, modern breeding technologies, sustainable agronomy, and socio-economic perspectives, this review presents a comprehensive framework for conserving and utilizing wheat landrace diversity to accelerate the development of climate-resilient wheat cultivars and strengthen global food and nutritional security under increasingly unpredictable climatic conditions.

## 1 Introduction

Wheat (Triticum spp.) is one of the world’s most important cereal crops, providing approximately 20% of the calories and protein consumed by the global population. Its remarkable adaptability to diverse agroecological conditions has enabled cultivation across temperate, Mediterranean, and dryland environments, making wheat a cornerstone of global food and nutritional security (Singh et al., 2023). In many arid and semi-arid regions, wheat remains a staple crop that supports both rural livelihoods and national food systems. However, the growing global population and increasing pressure on agricultural resources continue to raise concerns about the long-term sustainability of wheat production (Al-Khatib et al., 2023).

Climate change has emerged as one of the greatest challenges facing wheat cultivation. Rising temperatures, irregular rainfall patterns, prolonged drought periods, and increasing soil salinity are already affecting wheat-growing regions worldwide. These challenges are particularly severe in arid and semi-arid environments, where water availability is naturally limited and crops are frequently exposed to multiple stresses simultaneously (Hossain et al., 2021). Recent projections suggest that the frequency and intensity of heatwaves and drought events will continue to increase, posing substantial risks to wheat productivity, yield stability, and grain quality (Senapati et al., 2026).

Over the past century, remarkable advances in wheat breeding have contributed significantly to global food production. The development of high-yielding cultivars during the Green Revolution improved productivity and helped meet the food demands of a rapidly growing population (Singh et al., 2023). However, intensive selection for yield and uniformity has also reduced the genetic diversity present in modern wheat cultivars. This process, often referred to as genetic erosion, has narrowed the pool of adaptive traits available for coping with environmental stresses. As climate variability increases, concerns are growing regarding the ability of genetically uniform cultivars to maintain stable performance under increasingly unpredictable conditions (Malek et al., 2022).

In contrast, wheat landraces represent a valuable reservoir of genetic and phenotypic diversity. These locally adapted populations have evolved through centuries of natural selection and farmer-driven selection under diverse environmental conditions (Pramanik and Debnath, 2024). Unlike modern cultivars, which are typically bred for broad adaptation and high-input production systems, landraces have been shaped by specific local climates, soils, management practices, and cultural preferences. As a result, many landraces possess adaptive characteristics that enable them to persist in harsh environments where water scarcity, high temperatures, poor soil fertility, and biotic pressures frequently limit crop performance (Hossain et al., 2021).

Interest in wheat landraces has increased considerably in recent years as researchers seek new sources of resilience for climate-smart agriculture. Beyond their value as genetic resources for breeding programs, landraces provide important insights into the ecological, physiological, and evolutionary processes that contribute to long-term adaptation in challenging environments. Their continued cultivation in marginal agricultural systems also highlights their practical importance for maintaining food production under conditions where modern cultivars may perform inconsistently (Marone et al., 2021).

Previous reviews have established the importance of wheat landraces as reservoirs of genetic diversity and have examined their contribution to wheat improvement, stress adaptation, and the identification of useful traits and alleles (Adhikari et al., 2022). Other reviews have focused more specifically on individual components of climate-resilient wheat improvement, including abiotic-stress responses, molecular and omics-assisted approaches, and emerging breeding technologies. However, these dimensions are often considered separately, leaving a need to connect the evolutionary and farmer-mediated selection of wheat landraces with the mechanisms underlying their adaptive responses and, importantly, with their subsequent performance and utilization under field conditions.

The distinctive contribution of this review is to examine wheat landrace resilience as a connected process from adaptation to deployment. Specifically, it links the evolutionary history and diversity of landraces with physiological and biochemical responses, hormonal and molecular regulation, root–rhizosphere interactions, and the maintenance of agronomic and grain-quality traits under environmental stress. These biological responses are subsequently connected with multi-omics-based trait discovery, breeding applications, climate-adapted agronomic management, and the conservation and deployment of landrace diversity. Particular emphasis is placed on arid and semi-arid environments, where drought, heat, salinity, and their combinations impose strong and often simultaneous selection pressures. Rather than treating these components as independent attributes, this review evaluates their connections across biological and translational scales, providing a basis for identifying where evidence for landrace resilience is well established, where important uncertainties remain, and which adaptive characteristics offer the greatest potential for climate-resilient wheat improvement.

## 2 Evolutionary origins and environmental adaptation of wheat landraces

### 2.1 Centers of diversity and origin of arid-adapted wheat landraces

The diversity of wheat landraces observed today is the result of thousands of years of domestication, migration, environmental adaptation, and farmer selection. Following the domestication of wheat in the Fertile Crescent approximately 10, 000 years ago, cultivated forms gradually spread across a wide range of environments extending from the Mediterranean basin to Central and South Asia. During this expansion, wheat populations encountered contrasting climatic conditions, soil types, and farming systems, leading to the development of locally adapted landraces with distinct agronomic and morphological characteristics (Zencirci et al., 2021).

The Fertile Crescent, encompassing parts of present-day Iraq, Syria, Türkiye, Lebanon, Jordan, and Iran, remains one of the most important centers of wheat diversity. This region served as the primary center of domestication for both einkorn and emmer wheat and continues to harbor extensive genetic variation. The combination of diverse landscapes, seasonal rainfall patterns, and traditional farming practices contributed to the persistence of numerous locally adapted populations (Zaharieva et al., 2010; Zair, 2020).

Anatolia represents another important center of wheat diversity. The region contains a mosaic of climatic zones ranging from Mediterranean coastal environments to dry inland plateaus. Such environmental heterogeneity has favored the development of landraces adapted to varying levels of rainfall, temperature, and soil fertility. Several traditional wheat populations from Anatolia are still cultivated under low-input conditions and are recognized as valuable sources of adaptive genetic variation (Morgounov et al., 2016).

In North Africa, wheat cultivation has historically been practiced under rainfed conditions characterized by irregular precipitation and recurrent drought (Tidiane Sall et al., 2019). Countries such as Morocco, Algeria, Tunisia, and Egypt possess a rich collection of durum and bread wheat landraces that have persisted under challenging environmental conditions for centuries. These landraces form an important component of traditional farming systems and continue to contribute to local food security (Tidiane Sall et al., 2019).

Ethiopia is recognized as a secondary center of diversity for wheat. Geographic isolation, diverse agroecological zones, and long-standing traditional farming practices have contributed to the development of unique landrace populations. Ethiopian wheat landraces possess considerable genetic variation and have attracted significant interest for their potential use in crop improvement programs (Teklu and Hammer, 2006).

South Asia also contains valuable wheat landrace resources. Traditional wheat populations cultivated across India, Pakistan, Afghanistan, and neighboring regions have been exposed to variable rainfall patterns, high temperatures, and diverse soil conditions. Long-term adaptation to these environments has resulted in a broad range of locally adapted genotypes maintained through farmer-managed seed systems (Chatrath et al., 2007).

Although often overlooked, the Arabian Peninsula and surrounding regions also possess a history of wheat cultivation. Traditional wheat-growing areas in Yemen and Oman have maintained local landraces adapted to dry environments, limited water availability, and marginal soils. Interest in documenting and conserving these genetic resources has increased in recent years as countries in the Gulf region seek climate-resilient crops capable of supporting future food security initiatives. For countries such as the United Arab Emirates, regional wheat germplasm therefore represents a potentially valuable resource for investigating adaptation to arid environments, although its climate-resilience value requires direct evaluation under representative field conditions (Farooq, 2025).

The geographic distribution of wheat landraces across arid and semi-arid regions highlights the remarkable diversity that has evolved under contrasting environmental conditions (**Table 1**). Despite sharing a common domestication history, landraces from different regions exhibit distinct characteristics that reflect long-term adaptation to local climates, production systems, and farmer preferences. For example, einkorn and emmer populations originating from the Fertile Crescent are recognized for their adaptation to marginal environments, whereas durum wheat landraces from North Africa have been maintained under rainfed conditions characterized by limited and variable rainfall (Tidiane Sall et al., 2019). Similarly, traditional wheat populations from Ethiopia, South Asia, and the Arabian Peninsula represent valuable reservoirs of locally adapted genetic diversity (Morgounov et al., 2021). Collectively, these examples illustrate how environmental conditions and human selection have shaped the diversity of wheat landraces across different agroecological zones, providing a foundation for understanding the processes of local adaptation discussed in the following section. The broad geographic distribution of wheat landraces reflects long-term evolutionary adaptation to diverse agroecological conditions rather than random dispersal. Continuous exposure to contrasting climatic regimes, soil characteristics, and farming systems has generated locally adapted populations with distinct genetic and phenotypic characteristics. Consequently, the remarkable environmental heterogeneity across wheat-growing regions has served as a natural evolutionary laboratory, shaping the adaptive diversity that underpins the resilience of wheat landraces to multiple abiotic stresses.

**Table 1:** Representative wheat landraces and traditional wheat species cultivated across arid and semi-arid regions and their adaptation characteristics.

Species/landrace	Region/country	Typical annual rainfall (mm)	Production environment	Distinctive characteristics	Reported climate-resilience potential
Einkorn (*Triticum monococcum*)	Türkiye, Greece, Balkans	300–600	Marginal dryland farming systems	Hulled grain, early maturity, low-input adaptation	Adaptation to marginal, low-input environments (Zaharieva and Monneveux, 2014)
Emmer (*Triticum dicoccum*)	Fertile Crescent, Ethiopia	250–600	Rainfed semi-arid regions	Robust growth, broad genetic diversity	Potential drought- and disease-resilience traits (Biradar et al., 2022)
Durum landraces (*Triticum durum*)	Morocco, Algeria, Tunisia, Egypt	200–450	Mediterranean dryland agriculture	Stable grain production under limited rainfall	Potential drought- and heat-adaptive traits (Ayed et al., 2021)
Bread wheat landraces (*Triticum aestivum*)	Afghanistan, Iran, Turkey	250–500	Traditional rainfed systems	Wide phenotypic variation and local adaptation	Stress-adaptive genetic diversity (Morgounov et al., 2021)
Ethiopian wheat landraces	Ethiopia	400–1200	Highland and mixed farming systems	Unique genetic background and farmer-selected diversity	Potential source of adaptive alleles (Eticha et al., 2006)
Indian wheat landraces	India	250–600	Semi-arid and irrigated systems	Adaptation to terminal heat and variable rainfall	Potential source of heat-adaptive traits (Routray et al., 2007)
Pakistani wheat landraces	Pakistan	200–500	Arid and semi-arid regions	Adaptation to water scarcity and high temperatures	Potential drought-resilience traits (Gulnaz et al., 2025)
Afghan wheat landraces	Afghanistan	200–400	Mountainous dryland agriculture	Adapted to harsh climatic conditions	Potential for climate-adaptation breeding (Tehseen et al., 2021)
Omani traditional wheats	Oman	<250	Oasis and terrace farming systems	Adapted to extreme water limitation	Potential arid-adaptation traits (Farooq, 2025)
Israeli–Palestinian	Israel, Palestine	<300	Mountain and dryland agriculture	Long-term adaptation to low rainfall environments	Potential for dryland breeding (Frankin et al., 2020)
Spelt (*Triticum spelta*)	Central and Eastern Europe, Middle east	400–800	Low-input farming systems	Stress tolerance and grain quality attributes	Potential resilience and nutritional traits (Serhii et al., 2018)

### 2.2 Environmental filtering and local adaptation

The distribution of wheat landraces across arid and semi-arid regions did not occur randomly. Instead, environmental conditions acted as long-term selective forces that shaped the genetic composition of local populations. Over many generations, factors such as rainfall availability, temperature patterns, soil characteristics, and seasonal variability influenced which genotypes survived, reproduced, and were retained within farming systems (Babalola et al., 2025). This process, often described as environmental filtering, contributed to the emergence of landraces specifically adapted to local environmental conditions. As wheat populations spread into new environments, individuals possessing characteristics favorable for survival under those conditions were more likely to persist. Consequently, geographically separated landraces often developed distinct characteristics despite sharing a common ancestral origin (Marone et al., 2021).

In addition to environmental filtering, genotype × environment (G × E) interactions contribute substantially to adaptive variation among wheat landraces. Direct evaluations of wheat landraces under contrasting drought, heat, and favourable conditions have demonstrated significant variation in agronomic and phenological responses across environments (Sareen et al., 2014). Such variation indicates that the expression of adaptive traits is strongly influenced by environmental context, with some landraces showing greater stability or stress adaptation than others. These findings emphasize the importance of evaluating landrace performance across multiple environments rather than assuming that local origin alone predicts stress resilience. The resulting diversity among wheat landraces therefore reflects both their evolutionary history and the cumulative effects of local environmental conditions over centuries of cultivation (Abu-Zaitoun et al., 2018). However, historical persistence in a particular environment should not itself be interpreted as evidence of tolerance to a specific stress, which requires direct experimental evaluation.

### 2.3 Farmer selection as a driver of diversity

In addition to natural selection, farmers played a central role in shaping wheat landrace diversity. For thousands of years, farming communities have continuously selected seed from plants that performed well under local conditions and met specific household needs. This process occurred long before the development of formal breeding programs and remains one of the key factors responsible for the diversity observed among traditional wheat populations (Dias et al., 2024). Farmer selection often extended beyond grain yield. Characteristics such as grain quality, straw production, maturity period, storage performance, and suitability for local foods frequently influenced seed selection decisions. As a result, wheat landraces evolved as complex populations capable of satisfying both environmental and socio-economic requirements (Zencirci et al., 2021). Traditional seed exchange networks further contributed to the development and maintenance of diversity. The movement of seed among neighboring communities allowed genetic material to be shared while local selection continued to shape adaptation. Through this interaction between human management and environmental pressures, wheat landraces became dynamic populations that reflected both ecological conditions and cultural practices (Marone et al., 2021).

### 2.4 Conserving evolutionary potential in wheat landraces

The conservation of wheat landraces has become increasingly important as agricultural modernization and the widespread adoption of improved cultivars continue to reduce the cultivation of traditional varieties. Loss of landraces can result in the disappearance of valuable genetic resources that may be important for future crop improvement and climate adaptation efforts (Adhikari et al., 2022). Two main approaches are currently used for conserving wheat landrace diversity. *In situ* conservation involves maintaining landraces within their traditional farming environments, allowing them to continue evolving under local conditions. This approach preserves not only genetic diversity but also the ecological and cultural knowledge associated with traditional cultivation systems (Bellon et al., 2017).

*Ex situ* conservation relies on the storage of genetic resources in seed banks and germplasm collections. International and national genebanks currently maintain thousands of wheat accessions collected from diverse regions worldwide. These collections provide an important safeguard against genetic loss and serve as valuable resources for research and breeding programs. Both conservation approaches are complementary. While *ex situ* collections ensure long-term preservation of genetic resources, *in situ* conservation maintains the evolutionary processes that generate and sustain adaptation. Together, these strategies are essential for protecting wheat landrace diversity and ensuring its availability for future agricultural challenges (Rajpurohit and Jhang, 2015). Beyond preserving genetic resources, conservation strategies should aim to maintain the evolutionary processes that generate adaptive diversity. *In situ* conservation enables wheat landraces to continue responding to changing environmental conditions, while *ex situ* collections safeguard unique alleles for future research and breeding. Conserving both genetic diversity and evolutionary potential will be essential for developing climate-resilient wheat cultivars under increasingly unpredictable environmental conditions.

## 3 Adaptive strategies for environmental resilience in wheat landraces

persistence of wheat landraces across arid and semi-arid environments reflects long-term natural and farmer-mediated selection under locally variable climatic and edaphic conditions. Recurrent exposure to drought, high temperatures, nutrient limitations, salinity, and irregular rainfall has contributed to substantial genetic and phenotypic diversity within traditional wheat populations (Abu-Zaitoun et al., 2018; Babalola et al., 2025). This diversity provides a basis for identifying adaptive traits associated with performance under environmental stress, although the expression and effectiveness of these traits vary among landraces and across environments.

Rather than functioning as isolated mechanisms, these adaptive traits operate as integrated adaptive strategies, in which morphological, physiological, biochemical, and developmental responses are coordinated to optimize plant performance under adverse conditions. For example, phenological adjustment, efficient water acquisition, cellular protection, and post-stress recovery function together to minimize stress damage while maintaining growth and reproductive success. This integrated perspective reflects the complex nature of environmental adaptation, where multiple biological processes interact simultaneously in response to changing environmental conditions instead of acting independently (Bursakov et al., 2025). However, comparable studies directly examining such combined-stress responses in wheat landraces remain limited. This evidence gap is important because mechanisms identified in modern cultivars cannot automatically be assumed to operate similarly in genetically diverse landrace populations.

Under field conditions, wheat is frequently exposed to multiple environmental stresses rather than to individual stresses in isolation. Drought may coincide with elevated temperature, salinity, nutrient limitation, or other environmental constraints, and experimental studies in modern bread wheat have shown that combined stresses can produce physiological, metabolic, and molecular responses that differ from those observed under individual stresses (Galani et al., 2022; Da Ros et al., 2023). However, comparable studies directly examining such combined-stress responses in wheat landraces remain limited. This distinction is important because responses characterized in modern cultivars cannot automatically be interpreted as evidence of landrace-specific resilience.

Based on these responses, environmental resilience in wheat landraces can be examined through four major adaptive strategies: escape, avoidance, tolerance, and recovery. These strategies are not necessarily complementary or equally beneficial under all conditions; their contribution depends on stress timing, intensity, duration, and the target environment. For example, traits that promote stress escape may reduce exposure to terminal drought or heat but can constrain growth duration and yield potential, whereas water-conserving avoidance responses may protect plant water status at the expense of carbon assimilation. Accordingly, resilience should be considered as an environment-dependent balance among adaptive strategies rather than the accumulation of individual stress-response traits.The conceptual framework illustrating how escape, avoidance, tolerance, and recovery operate as complementary adaptive strategies to enhance environmental resilience in wheat landraces is presented in **Figure 1**.

**Figure 1:**
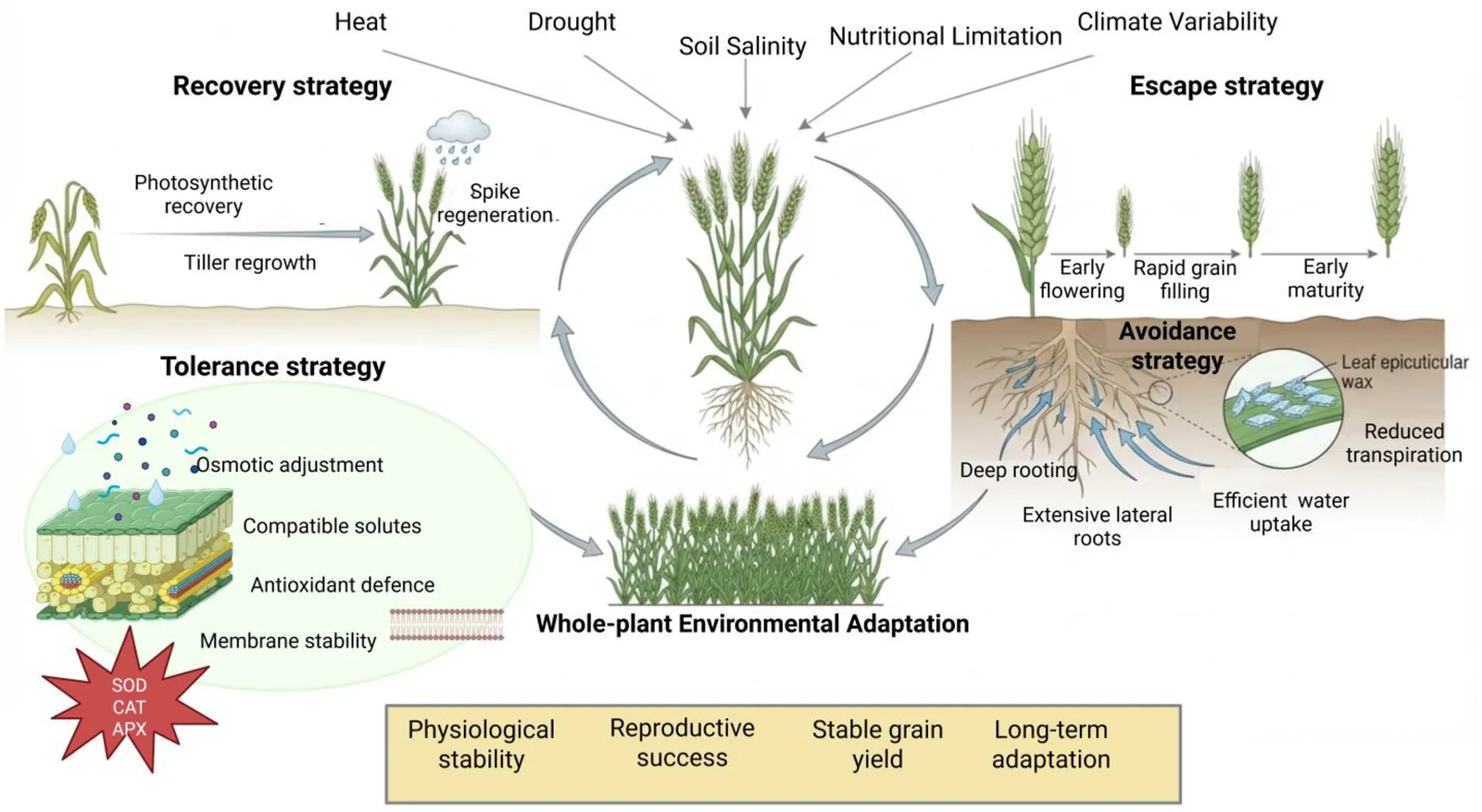
Adaptive strategies contributing to environmental resilience in wheat landraces cultivated under arid and semi-arid conditions.

### 3.1 Escape strategy

Stress escape is a phenological strategy in which wheat reduces exposure of stress-sensitive developmental stages to adverse conditions by adjusting the timing of its life cycle. Under environments characterized by terminal drought or late-season heat, earlier flowering and maturity can allow reproductive development to occur before stress reaches its greatest intensity. Unlike physiological tolerance, therefore, escape does not necessarily increase the plant’s capacity to withstand severe stress; rather, it reduces the temporal overlap between susceptible developmental stages and the period of maximum environmental stress (Shavrukov et al., 2017).

Direct evidence from wheat landraces supports the importance of phenological variation in this strategy, while also demonstrating that it is not a universal characteristic of landraces. Analysis of 645 Chinese wheat landraces showed that early-flowering accessions were generally more drought resistant than later-flowering accessions, linking variation in flowering time with drought response (Lin et al., 2019). Similarly, eastern Mediterranean durum wheat landraces flowered earlier and carried a high frequency of alleles associated with shorter developmental cycles compared with western Mediterranean landraces. These differences were interpreted as adaptation to the warmer, drier environments of the eastern Mediterranean, where earlier development can reduce exposure to post-flowering heat and water scarcity (Soriano et al., 2018). Importantly, however, eastern Mediterranean landraces did not flower significantly earlier than modern durum cultivars, demonstrating that early phenology is not an intrinsic advantage of landraces over improved germplasm.

The adaptive value of escape therefore depends on environmental context rather than on earliness alone. Early flowering can be advantageous when terminal stress is predictable because it reduces exposure during reproductive development; however, shortening the vegetative period can also restrict the time available for biomass accumulation and resource acquisition when water remains available or the growing season is longer (Shavrukov et al., 2017). Moreover, Mediterranean landrace evidence indicates that phenological adaptation is linked with other yield-formation traits: eastern landraces combined earlier flowering with longer grain-filling duration and greater grain number, but produced lighter grains than western landraces (Soriano et al., 2018). Thus, escape should not be viewed simply as an early-flowering phenotype, but as part of an environment-specific combination of phenological and yield-forming traits whose value depends on the timing and severity of stress.

### 3.2 Avoidance strategy

While escape reduces temporal exposure to stress, avoidance mechanisms reduce the intensity of stress experienced by maintaining access to water or limiting water loss during plant development. Root system architecture is therefore an important component of drought avoidance, but available evidence does not support a single universally advantageous root phenotype among wheat landraces. Direct comparison of durum wheat landraces with their wild relatives revealed contrasting rooting strategies, with the landraces tending toward greater exploration of deeper soil layers, a configuration potentially favourable for accessing stored subsoil water when surface moisture becomes limiting (Nakhforoosh et al., 2021). However, this experiment was conducted under well-watered conditions, and therefore demonstrates variation in root architecture rather than direct evidence of superior drought performance.

Substantial root diversity has also been demonstrated directly among Mediterranean bread wheat landraces. Analysis of 170 landraces identified variation in seminal root architecture and genomic regions associated with root traits, with several regions co-localizing with those associated with grain yield under rainfed field conditions (Rufo et al., 2020). Nevertheless, the relationship between root architecture and field performance is strongly environment-dependent. In a mixed panel of durum wheat germplasm, deeper-rooted classes achieved a 28–42% grain-yield advantage in environments with limited moisture, whereas the deepest-rooted types showed a 20–40% yield penalty relative to shallow-rooted types under moisture-rich conditions (El Hassouni et al., 2018). These findings indicate that deeper rooting should not be regarded as inherently superior; rather, its adaptive value depends on the depth and temporal availability of soil water and on the balance between resource acquisition and the carbon investment required for root growth. Consequently, root traits derived from landraces are most valuable for breeding when matched to the water regime of the target environment rather than selected as universally beneficial drought-resilience traits.

Above-ground physiological responses provide an additional component of stress avoidance by regulating water loss during periods of limited water availability. In wheat, stomatal closure reduces transpiration and can help maintain plant water status as soil moisture declines, while changes in leaf architecture and surface characteristics may further reduce water loss and heat load (Kulkarni et al., 2017). However, these responses involve an important physiological trade-off: prolonged or excessive stomatal closure restricts CO_2_ uptake and photosynthetic carbon assimilation, potentially reducing biomass accumulation and grain production. Although such water-conserving mechanisms are well established in wheat, direct comparative evidence demonstrating that they are consistently enhanced in wheat landraces relative to modern cultivars remains limited. Avoidance should therefore not be interpreted as maximal water conservation, but as the capacity to balance water acquisition and water loss while sustaining carbon assimilation during stress. Together with the root-system evidence described above, this indicates that the breeding value of avoidance traits depends on their ability to maintain this balance under the soil-water distribution, stress duration, and evaporative demand of the target environment.

### 3.3 Tolerance strategy

When environmental stress cannot be escaped or sufficiently avoided, tolerance mechanisms enable wheat to maintain cellular integrity and metabolic function despite declining tissue water status, elevated temperature, or oxidative stress. Osmotic adjustment and redox regulation are important components of this response, but evidence from wheat landraces indicates considerable genotypic variation in their contribution to stress tolerance. In eight durum wheat landraces exposed to moderate and severe drought, proline, soluble sugars, phenolic compounds, antioxidant activity, membrane stability, and plant water status responded differently among genotypes (Chaouachi et al., 2023). Notably, the landrace showing the greatest proline accumulation was not among those identified as the most drought tolerant, whereas the more tolerant landraces maintained a broader combination of favourable physiological and biochemical responses. Thus, accumulation of individual osmolytes should not be interpreted in isolation as evidence of superior tolerance.

A similar distinction emerges for antioxidant defence. Drought-tolerant landraces can exhibit stronger enzymatic and non-enzymatic protection against oxidative damage, but the adaptive significance lies in limiting cellular injury rather than simply increasing antioxidant activity. Direct comparison of the drought-tolerant wheat landrace Bolani with the drought-sensitive cultivar Sistan showed that Bolani maintained lower H_2_O_2_ accumulation, lipid peroxidation and electrolyte leakage while exhibiting stronger antioxidant and metabolic adjustments under drought (Naderi et al., 2020). In the durum landrace panel examined by Chaouachi et al. (2023), antioxidant responses likewise differed among genotypes, with some tolerant landraces combining greater catalase and peroxidase activities with reduced oxidative damage. Collectively, these results indicate that effective biochemical tolerance is better characterized by coordinated maintenance of redox homeostasis, membrane stability and physiological function than by the magnitude of any single stress-responsive metabolite or enzyme.

The importance of such coordination becomes greater when stresses occur simultaneously. Evaluation of Omani wheat landraces under drought, heat, and their combination demonstrated variation in physiological and biochemical responses together with differences in grain-yield performance, showing that tolerance identified from individual biochemical traits must ultimately be interpreted in relation to agronomic performance under the relevant stress environment (Farooq, 2023). At the molecular level, transcriptomic analysis of climate-adapted wheat landraces exposed to early heat stress further revealed extensive differential gene expression and identified regulatory networks associated with the heat-stress response, including candidate regulators linked to heat-shock-protein-mediated protection (Barratt et al., 2024). However, transcriptional activation alone does not establish field-level tolerance, and candidate regulatory mechanisms require validation across developmental stages and field environments.

Tolerance in wheat landraces should therefore be viewed as a coordinated and genotype-dependent capacity to limit cellular damage while sustaining physiological and reproductive function, rather than as the accumulation of individual biochemical markers. This distinction is particularly important for breeding: proline accumulation, antioxidant activity, or stress-responsive gene expression may provide useful screening indicators, but their value depends on whether they are associated with maintenance of biomass, fertility, and grain yield under the target stress regime. Consequently, landrace-derived tolerance traits should be prioritized only after their physiological effects are linked with stable agronomic performance across relevant environments.

### 3.4 Recovery strategy

Recovery represents the capacity to restore growth and physiological function after environmental stress is relieved and is particularly relevant where drought occurs intermittently rather than persisting until crop maturity. Unlike avoidance and tolerance, which operate during stress exposure, recovery determines the extent to which stress-induced reductions in plant function can be reversed after favourable conditions return. Experimental evidence in wheat shows that this capacity depends strongly on stress severity and developmental stage. Following drought and subsequent rewatering, wheat cultivars exhibited recovery of photosynthesis, stomatal conductance, water status, membrane stability, and related physiological processes, but recovery was more complete following moderate than severe drought and differed between tillering and jointing stages (Abid et al., 2018). Importantly, the more drought-tolerant cultivar showed stronger recovery and smaller subsequent yield reductions, indicating that post-stress restoration can contribute to agronomic performance rather than merely plant survival.

Direct evidence for recovery in wheat landraces is considerably more limited and has primarily been examined during early establishment. Ahmadizadeh et al. (2012) evaluated 37 durum wheat landraces/accessions from Iran and Azerbaijan and demonstrated significant genotypic variation in germination recovery following drought stress under laboratory and greenhouse conditions. Recovery percentages under the two experimental conditions were positively correlated, and several genotypes were identified as potentially useful germplasm for drought-tolerance breeding. However, this study assessed recovery germination rather than restoration of physiological function in established plants, and therefore does not demonstrate that these landraces can recover photosynthesis, vegetative growth, or reproductive performance following drought later in development (Ahmadizadeh et al., 2012).

This distinction exposes an important gap in current landrace research. Although post-drought recovery of physiological function and yield formation has been experimentally demonstrated in wheat cultivars, comparable evidence across diverse wheat landraces and developmental stages remains scarce. Recovery should therefore not be assumed from drought tolerance measured during stress, because the capacity to withstand stress and the capacity to resume function after stress relief represent related but distinct components of resilience. Its agronomic value is also environment-dependent: rapid recovery may be particularly beneficial where drought is interrupted by rainfall or irrigation, whereas it offers less advantage under terminal drought when no substantial recovery period occurs before maturity. Future evaluation of wheat landraces should therefore incorporate stress–rewatering experiments that quantify restoration of photosynthesis, growth, tillering, reproductive development, and ultimately grain yield. Such measurements would determine whether landrace variation in recovery can provide practically useful traits for breeding wheat adapted to increasingly variable rainfall regimes.

The adaptive strategies summarized in **Table 2** demonstrate that environmental resilience in wheat landraces does not depend on a single trait, but on the context-dependent contribution of multiple mechanisms operating at different developmental stages. Escape, avoidance, tolerance, and recovery are not equally advantageous under all conditions, and their effectiveness depends on the timing, intensity, duration, and recurrence of environmental stress. Their breeding value therefore depends on whether particular combinations of adaptive traits translate into stable growth, reproductive success, and grain yield under the target environment. Among the environmental constraints affecting wheat production, heat stress is particularly important because it can disrupt plant growth, physiology, reproductive development, and grain quality across multiple stages of the crop life cycle. Examining how wheat landraces respond to elevated temperature therefore provides an important basis for identifying adaptive variation with potential value for climate-resilient wheat improvement (Yanagi, 2024).

**Table 2:** Adaptive strategies contributing to environmental resilience in wheat landraces: benefits, trade-offs, evidence strength, and breeding relevance.

Adaptive strategy	Representative landrace-associated traits	Primary adaptive benefit	Limitation or trade-off	Environmental context/breeding relevance	Evidence specifically from wheat landraces
Escape	Early flowering, early maturity, and adjustment of developmental duration	Reduces overlap of reproductive development with terminal drought or heat	Excessive earliness may shorten vegetative growth and restrict biomass accumulation; phenological advantage is not unique to landraces	Most valuable where terminal drought or heat is predictable; breeding requires matching phenology to the target growing season	Chinese wheat landraces show associations between earlier flowering and drought resistance, while Mediterranean durum landraces show geographically differentiated phenology (Lin et al., 2019; Soriano et al., 2018)
Avoidance	Variation in root depth and distribution; stomatal regulation and water-conserving shoot responses	Maintains water acquisition and/or reduces water loss as soil moisture declines	Deeper rooting is not universally advantageous and may be disadvantageous where water is concentrated near the surface; strong stomatal closure conserves water but restricts CO_2_ assimilation	Root architecture should be matched to soil depth and water distribution rather than selected universally for maximum depth	Direct landrace studies demonstrate substantial root architectural diversity; field evidence linking root depth with yield is derived partly from mixed durum germplasm (Nakhforoosh et al., 2021; Rufo et al., 2020; El Hassouni et al., 2018)
Tolerance	Osmotic adjustment, soluble sugars, antioxidant defence, maintenance of membrane and redox homeostasis, and heat-responsive regulatory networks	Limits cellular injury and maintains metabolic and physiological function after tissue stress develops	High accumulation of individual markers such as proline does not necessarily indicate greater tolerance; biochemical responses do not automatically translate into yield protection	Breeding value depends on association of cellular protection with maintained photosynthesis, reproductive function, and grain yield under the target stress	Direct landrace studies demonstrate genotype-dependent osmotic, antioxidant, metabolic, and molecular responses under drought, heat, and combined stresses (Naderi et al., 2020; Chaouachi et al., 2023; Farooq, 2023; Barratt et al., 2024)
Recovery	Recovery germination and restoration of physiological function following stress relief	Restores physiological activity and growth after stress is alleviated	Recovery depends on stress severity and developmental stage; mature-plant recovery remains poorly characterized specifically in landraces	Particularly relevant under intermittent drought followed by rainfall or irrigation; less beneficial under terminal stress with little opportunity for recovery	Direct landrace evidence currently demonstrates substantial variation in recovery germination, whereas detailed post-drought physiological recovery is better established in wheat cultivars (Ahmadizadeh et al., 2012; Abid et al., 2018)

## 4 Biological integration of environmental adaptation in wheat landraces

Environmental adaptation in wheat landraces cannot be explained by individual stress-tolerance traits acting in isolation. Instead, successful performance under drought, heat, salinity, and their combined occurrence reflects the continuous interaction of multiple biological processes operating across different levels of plant organization. Changes in plant water relations, carbon assimilation, cellular metabolism, antioxidant defence, hormonal signalling, and gene regulation do not occur independently; they are tightly interconnected and respond dynamically according to the type, intensity, and duration of environmental stress. Consequently, the ability of wheat landraces to remain productive under adverse conditions is better understood as the outcome of coordinated biological responses rather than the expression of a single favorable characteristic (Chaouachi et al., 2023; Tripathi et al., 2026).

Evidence accumulated from physiological, biochemical, and molecular investigations consistently shows that stress-adapted wheat landraces regulate multiple processes simultaneously to preserve plant function under changing environmental conditions. Comparative studies have demonstrated that tolerant landraces maintain more stable water status, sustain photosynthetic activity, regulate oxidative balance, and activate stress-responsive regulatory pathways more effectively than susceptible genotypes when exposed to drought, heat, or combined stresses (Farooq, 2023; Chaouachi et al., 2024; Li et al., 2026). Although these adaptive responses have often been described separately, increasing evidence suggests that their collective interaction ultimately determines plant performance. Examining these responses within an integrated biological context provides a clearer understanding of how wheat landraces maintain function across diverse environments and establishes the basis for the physiological, biochemical, and regulatory mechanisms discussed in the following sections.

### 4.1 Maintaining plant function

The ability of wheat landraces to withstand environmental stress depends first on maintaining normal plant function despite adverse external conditions. Before visible symptoms such as wilting or growth inhibition appear, plants perceive subtle changes in soil water availability, temperature, and ionic balance through specialised sensing mechanisms that initiate a cascade of physiological responses. These early signals trigger communication between roots and shoots, allowing the plant to adjust growth, resource allocation, and water use according to the severity and duration of stress. Rather than responding after damage has occurred, stress-tolerant landraces rapidly modify their physiological behaviour, enabling them to maintain functional stability under fluctuating environments (Kulkarni et al., 2017).

One of the important components of adaptation to water limitation is variation in root system architecture, which influences the ability of wheat to explore different soil layers and access available water. Direct evidence from durum wheat landraces and their wild relatives demonstrates contrasting rooting strategies rather than a universally superior root phenotype. Nakhforoosh et al. (2021) found that the evaluated landraces tended to allocate a greater proportion of their root systems to deeper soil layers, whereas the wild relatives showed greater contribution of lateral and nodal roots to soil exploration. Because these plants were evaluated under well-watered conditions, however, the observed differences demonstrate variation in root architecture rather than direct evidence of superior drought tolerance (Nakhforoosh et al., 2021). Substantial root diversity has also been identified among Mediterranean bread wheat landraces, with genomic regions associated with seminal-root traits partly co-localizing with regions associated with grain yield under rainfed field conditions (Rufo et al., 2020). The adaptive value of root architecture is therefore context-dependent: deeper soil exploration may improve access to stored water under some drought scenarios, but no single root configuration can be considered universally advantageous across contrasting soil and moisture environments. Landrace root diversity consequently represents a valuable breeding resource when specific root traits are matched to the water distribution and soil characteristics of the target production environment.The benefits of enhanced root development become evident through improved plant water relations. Efficient water acquisition allows wheat landraces to maintain higher relative water content, leaf water potential, and tissue hydration during periods of environmental stress. These responses minimise cellular dehydration while sustaining physiological processes required for continued growth. Studies on Tunisian durum wheat landraces showed that drought-tolerant genotypes such as Aouija, Biskri, and Hedhba maintained significantly higher relative water content and lower electrolyte leakage than susceptible genotypes under progressive water deficit, indicating a greater capacity to preserve plant water status (Chaouachi et al., 2023). Similar responses have been reported in Omani wheat landraces exposed to combined drought and heat stress, where improved water relations were associated with greater biomass retention and reduced yield losses under multiple stress conditions (Farooq, 2023). These findings suggest that efficient water acquisition and conservation operate together to delay physiological deterioration during prolonged environmental stress.

Maintenance of plant water status is closely linked to stomatal regulation. Under water deficit and elevated temperature, stomatal closure limits transpirational water loss while balancing the need for continued carbon dioxide uptake required for photosynthesis. The ability to regulate stomatal conductance according to changing environmental conditions contributes directly to intrinsic water-use efficiency and allows tolerant landraces to optimize water consumption without completely suppressing carbon assimilation. Rather than maintaining permanently closed stomata, experimental evidence from wheat cultivars demonstrates dynamic stomatal responses to changing water availability, allowing continued metabolic activity under moderate stress while preventing excessive dehydration during severe stress (Abid et al., 2018). This dynamic regulation represents an important physiological adjustment linking water conservation with continued plant productivity.

Photosynthetic performance represents another major determinant of adaptation because carbon assimilation directly influences biomass accumulation and grain production. Environmental stress disrupts chloroplast structure, accelerates chlorophyll degradation, reduces electron transport efficiency, and limits carbon fixation. However, tolerant wheat landraces consistently exhibit greater capacity to preserve photosynthetic activity during stress exposure. Experimental evaluation of Tunisian durum wheat landraces demonstrated that tolerant genotypes maintained higher chlorophyll fluorescence, greater dry matter accumulation, and lower hydrogen peroxide concentrations under drought, heat, and combined stress compared with susceptible cultivars (Chaouachi et al., 2024). Similar observations in experimental wheat cultivar studiesindicate that sustained photosynthetic efficiency contributes directly to improved growth and reproductive performance under multiple environmental constraints (Li et al., 2026). Rather than simply delaying leaf senescence, maintenance of photosynthesis allows continued energy production necessary to support protective metabolic processes throughout the stress period.

Closely associated with these responses is the preservation of membrane integrity. Cellular membranes represent one of the earliest targets of abiotic stress because of dehydration, high temperature, and oxidative damage alter membrane fluidity and permeability. Maintenance of membrane stability reduces electrolyte leakage, preserves compartmentalization, and supports continued transport of water, nutrients, and metabolites between cells. Comparative studies consistently report lower membrane injury and greater membrane stability in drought- and heat-tolerant wheat landraces than in susceptible genotypes, demonstrating that protection of cellular structures contributes significantly to sustained physiological performance during environmental stress (Dugasa et al., 2019; Chaouachi et al., 2024). Stable membranes also facilitate the efficient functioning of photosynthetic tissues and provide the structural basis upon which subsequent biochemical and molecular responses can operate.

Taken together, these observations indicate that successful adaptation in wheat landraces depends on the coordinated maintenance of whole-plant function rather than exceptional performance of any individual trait. Early stress perception initiates rapid physiological adjustments that optimise root growth, maintain water acquisition, regulate transpiration, preserve photosynthetic activity, and protect cellular membranes. These responses operate simultaneously and reinforce one another, enabling plants to sustain growth under drought, heat, salinity, and their combined occurrence. The preservation of plant function therefore represents the first level of adaptation, creating the physiological conditions required for the biochemical and molecular processes discussed in the following sections.

### 4.2 Preserving cellular homeostasis under environmental stress

While maintenance of whole-plant function enables continued growth during environmental stress, long-term survival ultimately depends on preserving cellular homeostasis. Abiotic stresses such as drought, heat, and salinity disrupt water balance, alter cellular osmotic potential, impair metabolic processes, and promote the excessive accumulation of reactive oxygen species (ROS), resulting in oxidative damage to proteins, lipids, and nucleic acids. Wheat landraces minimise these detrimental effects through coordinated biochemical adjustments that stabilise intracellular conditions, allowing essential metabolic activities to continue even under prolonged or combined stress conditions. Rather than representing isolated defense mechanisms, these responses function collectively to maintain cellular integrity and sustain physiological performance (Tripathi et al., 2026).

One of the earliest biochemical responses to cellular dehydration is osmotic adjustment, which enables cells to retain water while maintaining turgor pressure and metabolic activity. This process is achieved through the accumulation of compatible solutes, small organic molecules that do not interfere with normal cellular metabolism even at high concentrations. Among these compounds, proline is one of the most extensively studied osmoprotectants in wheat. In addition to lowering cellular osmotic potential, proline stabilizes proteins and membranes, protects enzyme activity, and contributes to scavenging free radicals generated during stress. Proline accumulation increases in wheat under environmental stress, but its magnitude does not necessarily correspond directly with the degree of stress tolerance among landraces, indicating that its contribution should be interpreted together with other physiological and biochemical responses (Chaouachi et al., 2023, 2024). These observations indicate that osmotic regulation represents an important biochemical strategy for maintaining cellular function during environmental stress.

Alongside proline, soluble sugars and glycine betaine make important contributions to osmotic balance and cellular protection. Soluble sugars such as sucrose, glucose, and fructose act as osmolytes while simultaneously providing readily available energy for stress-responsive metabolism. They also contribute to membrane stabilization and protection of cellular macromolecules during dehydration. Glycine betaine performs complementary functions by preserving membrane structure, maintaining enzyme activity, and protecting the photosynthetic apparatus from stress-induced injury. The simultaneous accumulation of these compatible solutes can increase the capacity of wheat to tolerate fluctuating environmental conditions without disrupting normal cellular metabolism, particularly under combined drought and heat stress where osmotic imbalance becomes more severe (Abid et al., 2018; Li et al., 2026; Tripathi et al., 2026).

Maintenance of cellular homeostasis also requires efficient control of oxidative stress. Environmental stress frequently increases ROS production beyond the detoxification capacity of cells, leading to lipid peroxidation, membrane damage, and impairment of metabolic pathways. Wheat landraces counteract these effects through a highly coordinated antioxidant defence system comprising both enzymatic and non-enzymatic components. Antioxidant enzymes, including superoxide dismutase (SOD), catalase (CAT), ascorbate peroxidase (APX), guaiacol peroxidase (GPX), and glutathione peroxidase, rapidly convert highly reactive oxygen species into less harmful molecules, thereby limiting oxidative injury and preserving cellular integrity (Tripathi et al., 2026). Experimental studies on Tunisian durum wheat landraces demonstrated significantly higher catalase and glutathione peroxidase activities in drought-tolerant genotypes compared with susceptible cultivars under drought, heat, and combined stress conditions, reflecting a greater capacity to regulate oxidative balance (Chaouachi et al., 2023, 2024).

Non-enzymatic antioxidants provide an additional layer of cellular protection. Phenolic compounds and flavonoids accumulate in response to environmental stress and function as effective scavengers of reactive oxygen species while protecting cellular membranes from oxidative degradation. Beyond their antioxidant properties, these secondary metabolites contribute to membrane stabilization, maintenance of redox balance, and protection of photosynthetic tissues under adverse conditions. Increased concentrations of phenolics have been reported in drought-adapted wheat landraces together with enhanced antioxidant enzyme activities, suggesting that enzymatic and non-enzymatic defense systems operate cooperatively rather than independently to minimize oxidative damage during prolonged stress (Dugasa et al., 2019; Chaouachi et al., 2023). Such coordinated biochemical responses enable cells to preserve structural and metabolic integrity despite persistent environmental challenges.

The collective evidence indicates that preservation of cellular homeostasis is fundamental to environmental adaptation in wheat landraces. Osmotic regulation limits dehydration, compatible solutes maintain cellular hydration, antioxidant systems restrict oxidative injury, and secondary metabolites reinforce redox balance and membrane protection. These interconnected biochemical processes allow cells to remain metabolically active despite external stress, thereby supporting the physiological functions described in the previous section. Consequently, sustained whole-plant performance is not achieved solely through morphological or physiological adjustments but also through the continuous maintenance of a stable intracellular environment that buffers the effects of environmental stress.

### 4.3 Coordinating adaptive responses through regulatory networks

The physiological and biochemical adjustments described in the preceding sections do not operate independently but are coordinated through complex regulatory networks that integrate environmental signals with cellular responses. Rather than activating separate pathways for drought, heat, or salinity, wheat plants continuously exchange information between roots and shoots through interconnected signaling systems that synchronize growth, metabolism, and defense according to changing environmental conditions. This regulatory coordination enables wheat landraces to balance resource allocation between survival and continued development while avoiding unnecessary metabolic costs associated with prolonged stress responses (Tripathi et al., 2026). Consequently, successful adaptation depends not only on the presence of favorable physiological traits but also on the efficiency with which multiple signaling pathways communicate and function together.

Among these regulatory systems, phytohormones provide the primary framework for coordinating environmental responses. Abscisic acid (ABA) functions as one of the earliest signals following reductions in soil water availability, promoting stomatal regulation, osmotic adjustment, and activation of stress-responsive genes. However, ABA rarely acts alone. Its activity is continuously modified through interactions with ethylene, jasmonic acid (JA), and salicylic acid (SA), allowing plants to fine-tune growth, defense, and metabolic activity according to stress intensity and duration. Rather than functioning as independent signaling molecules, these hormones form an interconnected communication network that coordinates multiple adaptive processes simultaneously. Such hormonal crosstalk is particularly important under combined environmental stresses, where conflicting physiological demands require balanced regulation rather than activation of single stress-response pathways (Ahmad et al., 2025).

Hormonal signaling is subsequently translated into coordinated changes in gene expression through the activation of transcription factors and regulatory genes. Families such as DREB, NAC, WRKY, MYB, and bZIP regulate numerous downstream genes involved in osmotic adjustment, antioxidant metabolism, membrane protection, photosynthetic maintenance, and cellular repair. Because individual transcription factors frequently regulate multiple stress-responsive pathways, they enable wheat plants to respond rapidly to complex environmental conditions without relying on independent regulatory systems for each stress factor. This integrated mode of regulation provides the flexibility required to respond to drought, heat, salinity, and their combinations, reflecting the polygenic nature of environmental adaptation in wheat (Kulkarni et al., 2017; Khalid and Ashraf, 2025).

Recent transcriptomic investigations have further demonstrated that wheat landraces possess distinctive regulatory signatures associated with enhanced stress tolerance. Co-expression network analyses of diverse landrace collections identified candidate master regulators that were rapidly activated during early drought and heat exposure, suggesting that naturally adapted germplasm possesses highly coordinated regulatory networks capable of initiating protective responses before extensive physiological damage occurs (Abu-Zaitoun et al., 2018; Barratt et al., 2023a, 2023b). These studies identified several candidate regulators associated with thermotolerance, drought adaptation, and maintenance of photosynthetic performance, providing valuable insight into the molecular mechanisms that distinguish stress-adapted landraces from more susceptible genotypes. Complementary quantitative trait locus (QTL) analyses have likewise identified genomic regions controlling root architecture, water-use efficiency, phenological adaptation, and stress tolerance, confirming that environmental adaptation is governed by numerous interacting loci rather than single major genes (Abu-Zaitoun et al., 2018).

Increasing evidence indicates that regulatory coordination becomes even more complex under combined environmental stresses. Simultaneous exposure to drought and heat, or drought and salinity, activates signaling pathways that differ substantially from those induced by individual stresses, producing unique transcriptional and hormonal responses rather than additive effects. Studies evaluating combined stress responses in wheat have consistently reported greater disruption of physiological processes together with extensive reprogramming of gene expression, highlighting the importance of regulatory flexibility in maintaining adaptation under realistic field conditions (Chaouachi et al., 2023; Li et al., 2026; Tripathi et al., 2026). The ability of wheat landraces to integrate multiple environmental signals into coordinated physiological and biochemical responses therefore represents as an important component of their potential adaptation to complex field environments; however, direct comparative evidence demonstrating that regulatory integration is stronger in landraces than in modern cultivars remains limited.Taking together, the available evidence indicates that environmental adaptation in wheat landraces is governed by highly interconnected regulatory networks rather than isolated signaling pathways. Hormonal crosstalk coordinates whole-plant responses, transcription factors translate environmental signals into appropriate patterns of gene expression, and multiple regulatory loci collectively determine the capacity to respond to single and combined stresses. This hierarchical organization enables wheat landraces to synchronize physiological function with cellular homeostasis while maintaining sufficient developmental plasticity to survive under highly variable environments. Understanding these regulatory interactions provides the biological link between observable adaptive traits and the genomic resources that are increasingly being explored through multi-omics approaches, discussed in the following section. Collectively, these findings demonstrate that environmental adaptation in wheat landraces is governed by an integrated signaling network in which early stress perception, hormonal crosstalk, transcriptional regulation, and downstream physiological responses operate in a coordinated manner rather than as isolated processes. Recent multi-omics investigations indicate that these regulatory components operate as highly interconnected molecular networks rather than independent signalling pathways, providing a systems-level understanding of environmental adaptation. A conceptual representation of these interconnected mechanisms is presented in **Figure 2**.

**Figure 2:**
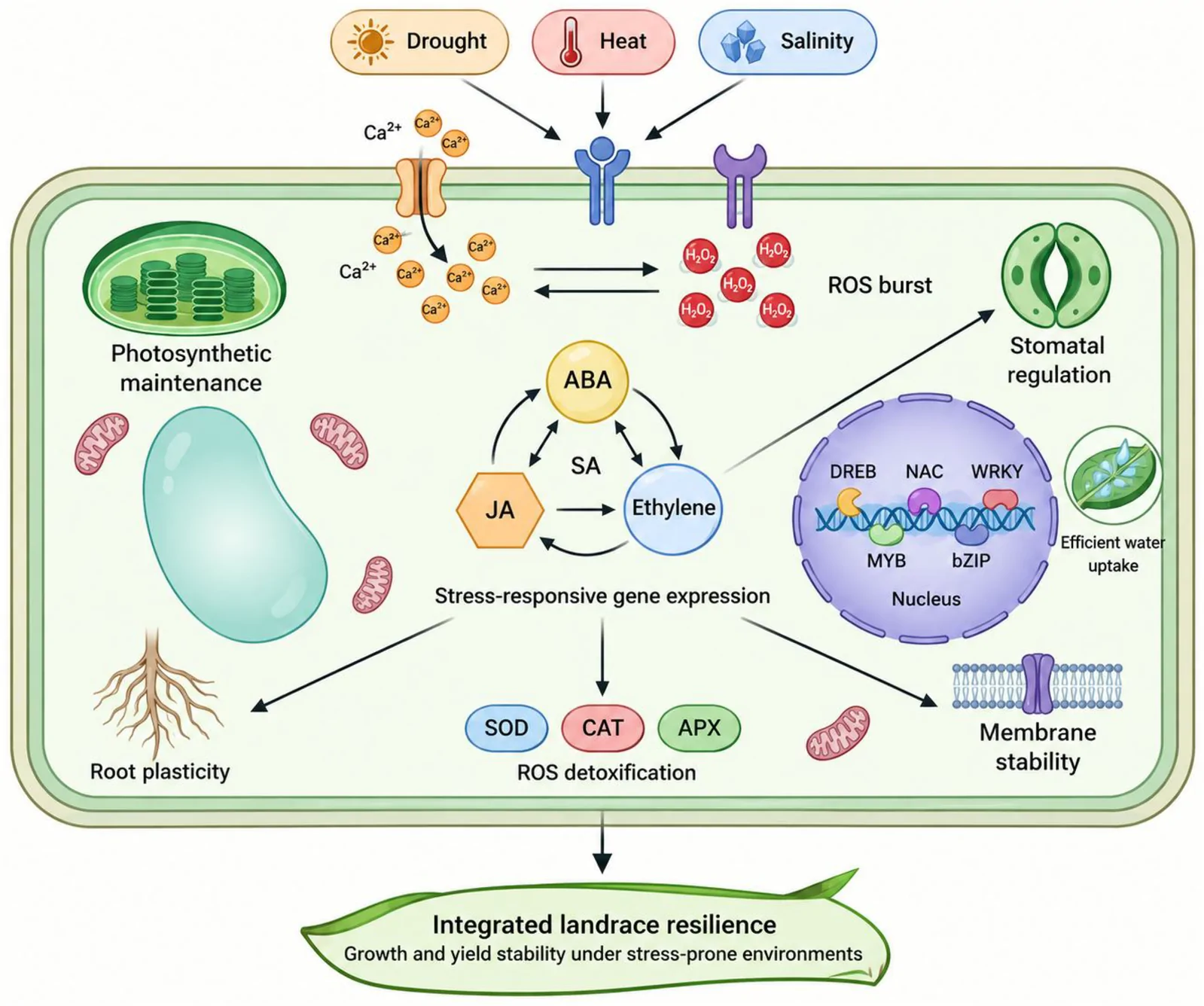
Conceptual model illustrating the integrated signaling network coordinating environmental adaptation in wheat landraces under combined abiotic stresses.

## 5 The hidden role of roots and the rhizosphere

Environmental resilience in wheat landraces is often discussed in terms of aboveground traits such as flowering time, leaf characteristics, and physiological stress responses. However, an equally important component of adaptation occurs belowground. The root system and the surrounding rhizosphere represent the primary interface between plants and soil, influencing water acquisition, nutrient uptake, microbial interactions, and stress signaling (Chen et al., 2022). Increasing evidence suggests that belowground processes play a central role in determining plant performance under environmental stress. For wheat landraces that have evolved under low-input and resource-limited conditions, root-associated traits and rhizosphere interactions may contribute substantially to long-term resilience. The interconnected roles of root architecture, rhizosphere processes, and plant-associated microorganisms in supporting resource acquisition, stress adaptation, and climate resilience are summarized in **Figure 3**.

**Figure 3:**
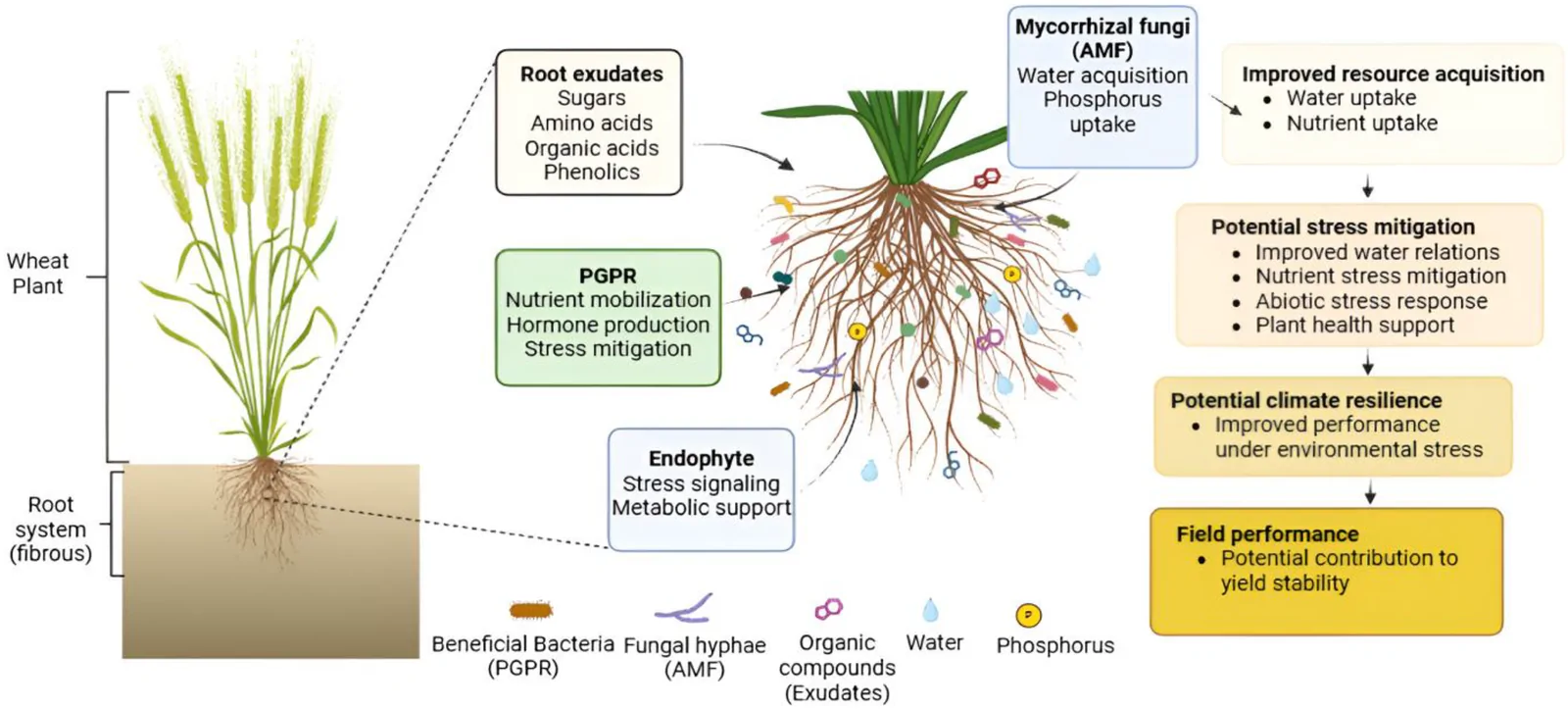
Conceptual framework of root–rhizosphere–microbiome interactions potentially contributing to environmental resilience in wheat landraces. Root architecture, root exudates, plant growth-promoting rhizobacteria (PGPR), endophytes, and arbuscular mycorrhizal fungi (AMF) can influence water and nutrient acquisition and stress responses.

### 5.1 Root architecture and resource acquisition

Root architecture determines how effectively plants explore soil resources and respond to environmental variability. While root traits have traditionally been evaluated in the context of drought adaptation, their influence extends beyond water acquisition alone. Root systems shape the physical and biological environment surrounding the plant and therefore influence multiple aspects of stress resilience (Chen et al., 2017).

Variation in root architecture affects both the spatial distribution of roots within the soil profile and the efficiency with which resources are captured. Root growth angle influences rooting depth, with steeper angles promoting deeper soil exploration and access to subsoil moisture, whereas shallower angles favor exploitation of nutrient-rich surface horizons. Deep-rooting genotypes can access water and nutrients located in deeper soil layers, while wider root systems with extensive lateral branching enhance exploration of surface soils where relatively immobile nutrients are often concentrated (Chen et al., 2017). Differences in root biomass allocation further influence the balance between root proliferation and aboveground growth, affecting overall resource-use efficiency under limiting conditions. Root density and branching patterns determine the volume of soil explored and can enhance nutrient interception, while root hairs increase absorptive surface area and improve access to phosphorus and micronutrients (Ruiz et al., 2018). However, these root strategies involve functional trade-offs rather than representing universally advantageous phenotypes: deeper soil exploration may favour access to stored subsoil water, whereas greater investment in shallow and lateral roots can improve exploitation of surface resources, making the adaptive value of root architecture dependent on soil depth, nutrient distribution, and the timing and severity of water limitation (Nakhforoosh et al., 2021; El Hassouni et al., 2018).

Evidence from tetraploid wheat landraces indicates substantial natural variation in root depth, growth angle, root system width, branching architecture, and biomass distribution, suggesting that root traits have been shaped by long-term adaptation to local environmental conditions. Such diversity provides a valuable resource for improving resource acquisition and stress tolerance in future breeding programs (Ruiz et al., 2018). Comparisons between historical and modern wheat germplasm further indicate that breeding has altered root system architecture over time, modifying traits such as root angle, depth distribution, and biomass allocation. These observations suggest that certain adaptive root characteristics present in traditional landraces may represent valuable genetic resources for improving resource acquisition and stress resilience under future climatic conditions (McGrail and McNear, 2021). However, these differences should not be interpreted as evidence that landrace root systems are universally superior to those of modern cultivars. Rather, the extensive root diversity retained within landrace germplasm provides a source of specific architectural traits that may be valuable when matched to the soil and water conditions of target production environments.

The importance of root traits extends beyond direct resource capture. Root architecture influences rhizosphere formation through the release of root exudates and the creation of distinct chemical and biological microenvironments. Consequently, root systems not only determine how plants access resources but also shape interactions with soil microorganisms that contribute to plant health and stress adaptation.

### 5.2 Rhizosphere processes and environmental adaptation

The rhizosphere is a dynamic zone of intense biological activity surrounding plant roots. Within this region, complex interactions occur among roots, soil particles, microorganisms, and soluble nutrients. These interactions strongly influence plant nutrition, water availability, and overall soil health.

Root exudates play a particularly important role in shaping rhizosphere processes. Plants release a wide range of organic compounds, including sugars, amino acids, organic acids, phenolics, and secondary metabolites, which influence nutrient availability and microbial activity. Through these exudates, plants actively modify their surrounding environment and create conditions that support resource acquisition under challenging conditions. Emerging evidence further suggests that plant genotype influences rhizosphere microbiome assembly. Comparative studies across wheat evolutionary lineages have shown that domestication and breeding are accompanied by shifts in rhizosphere microbial communities and their functional capacities, indicating that wheat evolutionary history and host genetic background can influence rhizosphere microbiome assembly (Sun et al., 2025).

Nutrient acquisition within the rhizosphere involves multiple biological and chemical processes. Microbial activity can increase nutrient solubility, mobilize phosphorus, enhance nitrogen cycling, and improve the availability of essential micronutrients. These functions become especially important in arid soils where nutrient availability is often restricted by low organic matter content and limited microbial activity. The composition and functional activity of rhizosphere microbial communities are further influenced by interactions among soil properties, management practices, and plant genotype. Under drought conditions, these factors collectively drive microbial adaptations that affect nutrient turnover, resource availability, and plant stress responses (Breitkreuz et al., 2021). However, evidence that these drought-responsive rhizosphere functions differ systematically between wheat landraces and modern cultivars remains limited, and the contribution of landrace-specific microbiome assembly to nutrient acquisition under water-limited conditions requires direct experimental validation.

The rhizosphere also contributes to plant water relations. Soil aggregation promoted by roots and microorganisms can improve water retention and enhance soil structure. In dry environments, such modifications may increase the efficiency of water capture and reduce the impact of transient drought periods. In addition to biological effects, root exudates can modify the hydraulic properties of the rhizosphere by influencing soil wettability, water retention, and hydraulic conductivity around roots. Evidence from maize landraces and modern cultivars demonstrates that root and rhizosphere traits can influence soil resource acquisition and drought responses, illustrating a mechanism that may also be relevant to belowground adaptation in wheat, although direct evidence in wheat landraces remains limited (Wild et al., 2024). Consequently, the rhizosphere should be viewed not merely as a zone of nutrient uptake but as a critical component of the plant adaptation system.

### 5.3 Microbiome-mediated resilience

The plant microbiome has emerged as an important factor influencing crop performance under environmental stress. Microbial communities associated with roots, internal tissues, and the rhizosphere contribute to nutrient acquisition, stress mitigation, pathogen suppression, and growth regulation. For wheat landraces that have coexisted with local soil communities over long periods, microbiome interactions may represent an important yet underexplored component of resilience, although direct evidence linking landrace-associated microbiome composition to superior stress performance remains limited.

Plant growth-promoting rhizobacteria (PGPR) enhance plant performance through multiple mechanisms, including improved nutrient availability, phytohormone production, root stimulation, and modulation of stress signaling pathways. Certain PGPR strains can also improve tolerance to drought, salinity, and heat stress. Recent studies suggest that beneficial microorganisms may confer protection not only through direct antagonism but also by reshaping the broader rhizosphere microbiome. Such microbiome-mediated resistance can alter community composition and functional interactions, in wheat, bacterial inoculation has been shown to restructure the rhizosphere microbiome and enhance resistance to a soil-borne pathogen, demonstrating the functional importance of microbiome manipulation, although this does not directly establish improved abiotic-stress tolerance in wheat landraces (Nishisaka et al., 2026).

Endophytic microorganisms provide an additional layer of support by colonizing internal plant tissues without causing disease. These microorganisms can influence plant metabolism, improve nutrient utilization, and contribute to stress adaptation through the production of bioactive compounds. Increasing evidence suggests that endophytic communities play important roles in helping plants cope with environmental fluctuations (Noman et al., 2021). Arbuscular mycorrhizal fungi represent one of the most extensively studied beneficial plant–microbe associations. By extending root absorptive capacity through extensive hyphal networks, mycorrhizal fungi enhance phosphorus uptake, improve water acquisition, and influence plant responses to multiple environmental stresses. In nutrient-poor and water-limited soils, these interactions can substantially improve plant performance in wheat, bacterial inoculation has been shown to restructure the rhizosphere microbiome and enhance resistance to a soil-borne pathogen, demonstrating the functional importance of microbiome manipulation, although this does not directly establish improved abiotic-stress tolerance in wheat landraces (Munir et al., 2022).

Microbiome-assisted approaches are increasingly being recognized as complementary components of climate-resilient wheat breeding, highlighting the need to integrate plant genetics with beneficial microbial associations in future crop improvement programs (Kumar et al., 2026). Despite growing recognition of plant-associated microbiomes, their diversity and functional roles in wheat landraces remain poorly understood compared with modern cultivars. Understanding microbiome recruitment and long-term plant–microbe co-adaptation therefore represents an important research priority, particularly in arid and semi-arid environments where microbial interactions may strongly influence crop resilience. Future studies should directly compare landraces and modern cultivars grown in common soils and under controlled drought, heat, and salinity treatments to determine whether landraces recruit distinct microbial communities and whether these communities causally improve stress tolerance and yield stability.

### 5.4 Towards an integrated belowground adaptation framework

Although root architecture, rhizosphere processes, and plant-associated microorganisms are commonly investigated as separate components of plant adaptation, increasing evidence indicates that they function as an integrated belowground system rather than as independent mechanisms. Root system architecture determines the spatial distribution of roots within the soil profile, while root exudates modify the surrounding chemical environment and selectively recruit beneficial microbial communities. These microorganisms subsequently influence nutrient cycling, water acquisition, phytohormone production, and stress-responsive metabolic processes, creating continuous feedback between the plant and its surrounding soil environment. Consequently, the adaptive capacity of wheat landraces may depend not only on favourable root traits but also on interactions among roots, the rhizosphere, and associated microbial communities that influence resource acquisition and environmental responses; however, the functional integration of these components has not yet been comprehensively demonstrated in wheat landraces (Devesa-Aranguren et al., 2026; Francomano et al., 2026).

This integrated perspective has led to the recognition of plants and their associated microbial communities as a functional holobiont, in which the host plant and its microbiome operate as a coordinated biological unit rather than as separate entities. Within this framework, environmental adaptation is viewed as the result of dynamic interactions among plant genetics, root architecture, microbial recruitment, and soil processes, enabling plants to respond more effectively to multiple and fluctuating environmental stresses. For wheat landraces that have may depend not only on favourable root traits but also on interactions among roots, the rhizosphere, and associated microbial communities that influence resource acquisition and environmental responses; however, the functional integration of these components has not yet been comprehensively demonstrated in wheat landraces (Francomano et al., 2026).

Collectively, current evidence supports a belowground adaptation framework in which root architecture determines the spatial domain of soil exploration, rhizosphere processes modify resource availability and root–soil interactions, and microbial communities can further influence nutrient acquisition and stress responses. Wheat landraces contain substantial root architectural diversity, but evidence directly linking this diversity to distinctive rhizosphere microbiomes and subsequently to improved stress resilience remains limited. The key research challenge is therefore to move from studying roots, rhizosphere processes, and microbiomes independently toward testing their functional interactions under common environmental conditions and determining whether particular combinations improve yield stability under stress. Integrating root phenotyping with microbiome profiling, environmental measurements, and multi-omics approaches could identify belowground trait–microbe combinations with practical value for breeding wheat adapted to arid and increasingly variable environments.

## 6 Grain quality, nutritional stability, and food security under climate stress

The value of wheat landraces extends beyond their ability to survive under adverse environmental conditions. While resilience is often evaluated in terms of plant survival and yield stability, grain quality and nutritional stability are equally important components of climate resilience because maintaining crop productivity without preserving nutritional value cannot ensure long-term food and nutritional security. Consequently, understanding whether and under which environmental conditions wheat landraces maintain grain quality and nutritional composition under stress is essential for assessing their contribution to future food and nutritional security (Kaplan Evlice, 2021).

### 6.1 Protein quality and grain composition

Protein content is one of the most important determinants of wheat grain quality. Environmental conditions strongly influence protein accumulation during grain development, and stress often affects the balance between grain yield and protein concentration. Under climate stress, maintaining gluten functionality and end-use quality is as important as increasing total protein. Drought and heat can reduce starch deposition and grain yield while increasing the relative concentration of grain protein, meaning that higher protein concentration under stress does not necessarily indicate improved grain quality or greater stress resilience (Al-Naggar et al., 2020).

Many wheat landraces possess relatively high grain protein content compared with modern high-yielding cultivars. Studies comparing landraces and commercial cultivars have identified substantial variation in grain protein concentration, bread-making quality, and nutritional characteristics, highlighting the importance of traditional germplasm as a source of quality-related traits (Heidari et al., 2016).

Beyond total protein concentration, grain quality is strongly influenced by the composition of storage proteins. Variations in glutenins and gliadins affect dough strength, elasticity, baking performance, and other processing characteristics. Wheat landraces harbor considerable diversity in these storage proteins, providing potentially useful variation in protein composition and processing quality that may complement modern breeding germplasm (Branlard et al., 2020). However, evidence that the generally high or diverse protein characteristics of wheat landraces translate into greater stability of protein composition and processing quality under drought or heat remains more limited than evidence for their baseline compositional diversity.

### 6.2 Micronutrient stability under environmental stress

Micronutrient deficiencies remain a major global health concern, affecting billions of people worldwide. Wheat serves as an important dietary source of minerals such as zinc and iron, particularly in regions where cereal-based foods constitute a large proportion of daily caloric intake. Maintaining micronutrient concentrations under environmental stress therefore has important implications for food security and human nutrition (Gupta et al., 2024).

Environmental stresses can influence mineral uptake, translocation, remobilization, and accumulation within developing grains. Reduced soil moisture, altered root activity, and disruptions in nutrient transport may limit micronutrient deposition during grain filling. However, substantial variation exists among wheat genotypes in their ability to acquire and maintain essential minerals under challenging environmental conditions. Studies conducted across multiple environments have demonstrated genotypic variation in grain nutritional and quality traits under drought and terminal heat stress, indicating that the effects of environmental stress on grain composition depend strongly on genotype × environment interactions (Kakanur Jagadeesha et al., 2024).

Considerable genetic variation for grain mineral composition has been reported in wheat landraces and wild relatives. Research on *Triticum dicoccoides* revealed significant variation and environmental stability for several mineral nutrients, demonstrating that genetically controlled variation in mineral accumulation and stability exists within the broader wheat gene pool (Gomez-Becerra et al., 2010). Such traits are particularly valuable under climate change scenarios where environmental variability may increasingly affect crop nutritional composition.

Numerous studies have identified wheat landraces with elevated concentrations of zinc, iron, and other essential minerals compared with modern cultivars (Heidari et al., 2016). These findings suggest that traditional germplasm represents an important reservoir of nutritional diversity that could support future biofortification efforts. The identification and conservation of micronutrient-rich landraces may therefore contribute not only to breeding for nutritionally resilient wheat but also to addressing hidden hunger and micronutrient deficiencies in vulnerable populations. However, higher baseline mineral concentrations in landraces should not be interpreted as evidence of greater micronutrient stability under drought or heat, because direct comparative studies evaluating mineral retention in landraces and modern cultivars across climate-stress environments remain limited. Thus, current evidence strongly supports wheat landraces as reservoirs of mineral diversity, whereas their capacity to maintain micronutrient concentrations more consistently than modern cultivars under drought, heat, and combined stresses remains an important question for direct comparative evaluation.

### 6.3 Phenolics and bioactive compounds

Phenolic compounds constitute an important group of secondary metabolites that contribute to both plant adaptation and human health. Wheat grains contain a diverse range of phenolic acids, flavonoids, and related bioactive compounds with antioxidant properties and potential health-promoting effects. These compounds occur in free, conjugated, and bound forms, with many concentrated in the outer grain layers (Saini et al., 2021).

Environmental stress often influences the synthesis and accumulation of phenolic compounds. Drought, heat, and other abiotic stresses can induce oxidative stress within plant tissues, stimulating secondary metabolic pathways involved in the production of protective antioxidants. Abiotic stress can alter phenolic metabolism and, under some conditions, increase the accumulation of particular phenolic compounds; however, the direction and magnitude of these responses depend on genotype, environmental conditions, and the nature and severity of the stress (Ma et al., 2021). Therefore, increases in individual phytochemicals under stress should not automatically be interpreted as improved overall grain quality, particularly when they occur alongside reductions in grain yield or other quality attributes.

Several studies have reported higher concentrations of phenolic compounds and other bioactive constituents in traditional wheat varieties and ancient wheat species compared with modern cultivars (Kowalska et al., 2024, 2026). Together with the broader compositional diversity reported in traditional wheat germplasm, these findings support the potential value of landraces as sources of biochemical diversity, but they do not by themselves demonstrate that landraces consistently contain higher concentrations of bioactive compounds than modern cultivars or maintain these compounds more effectively under climate stress. Variation in the abundance and composition of these bioactive compounds contributes directly to the antioxidant capacity and functional quality of wheat grain, discussed in the following section. Direct experiments comparing the stability of grain phenolic profiles in wheat landraces and modern cultivars exposed to the same drought, heat, or combined-stress environments are therefore needed to determine whether landrace biochemical diversity translates into greater functional-quality stability under climate stress.

### 6.4 Antioxidant capacity and functional quality

The accumulation of bioactive compounds is often reflected in the overall antioxidant capacity of wheat grain, an important indicator of nutritional and functional quality. Measurements based on assays such as DPPH, ABTS, and related methodologies have revealed substantial variation in antioxidant activity among wheat genotypes and *Triticum* species, reflecting differences in the concentration and composition of antioxidant metabolites accumulated during grain development (Ma et al., 2021).

Antioxidant capacity is influenced by a diverse range of grain constituents, including phenolic acids, flavonoids, carotenoids, tocopherols, and other reducing compounds. These metabolites help protect plant tissues against oxidative damage caused by reactive oxygen species (ROS), which increase under environmental stresses such as drought and heat. Environmental stress can alter antioxidant metabolism and the accumulation of these compounds; however, changes in antioxidant defence during plant stress do not necessarily translate directly into greater antioxidant capacity or functional quality of the harvested grain (Ma et al., 2021; Yiğit, 2025).

Genotype strongly influences the accumulation of antioxidant compounds. Comparative studies have demonstrated substantial differences among wheat cultivars, landraces, and related *Triticum* species in both the quantity and diversity of bioactive metabolites (Kowalska et al., 2024, 2026). Similarly, nutritional characterization of durum wheat landraces from Iran and Mexico revealed considerable variation in antioxidant-related compounds and nutritional quality traits, highlighting the value of traditional germplasm as a source of functional diversity for crop improvement, although this compositional variation does not itself demonstrate greater stability under environmental stress (Hernandez-Espinosa et al., 2020).

In addition to their nutritional significance, antioxidant-related traits may contribute to grain quality stability under environmental stress. Evidence from old wheat landraces and locally adapted varieties indicates genotypic variation in technological quality and responses to environmental stress, supporting the potential value of this germplasm for simultaneously investigating quality and resilience traits (Maravić et al., 2025). Thus, while direct evidence supports substantial antioxidant and functional diversity within wheat landrace germplasm, whether this diversity results in more stable antioxidant capacity than modern cultivars when both are exposed to the same climate stresses remains insufficiently resolved.

### 6.5 Yield–quality trade-offs under climate stress

A critical consideration in climate-resilient agriculture is the relationship between grain yield and grain quality. Historically, wheat improvement programs have focused primarily on increasing productivity. While this strategy has substantially enhanced food production, selection for high yield can be associated with trade-offs involving protein concentration, mineral composition, and other quality-related traits (Heidari et al., 2016; Fradgley et al., 2022).

Many wheat landraces exhibit lower maximum yield potential than modern cultivars can contain higher concentrations of particular protein, mineral, or other quality-related components.Higher grain yield can increase the proportion of carbohydrate relative to other grain constituents, contributing to dilution of protein, micronutrients, or bioactive compounds in some genotypes and environments (Fradgley et al., 2022).

The relationship between yield and quality is influenced by both genetic and physiological factors. Several quality-related traits and yield components are controlled by overlapping genetic regions, creating constraints that can make simultaneous improvement of productivity and grain quality challenging (Fradgley et al., 2022). In addition, grain number, grain weight, are determined during overlapping developmental phases, creating physiological trade-offs in the allocation of assimilates to developing grains. Environmental stress can further alter these source–sink relationships and grain-filling processes, with consequences for both productivity and grain composition (Vicentin et al., 2024).

Climate stress further complicates this balance by altering carbon and nitrogen partitioning during grain development. Heat and drought can shorten grain-filling duration and reduce starch accumulation, often lowering grain yield while increasing the relative concentration of protein because protein deposition and carbohydrate accumulation respond differently to stress (Al-Naggar et al., 2020).

From a climate adaptation perspective, maintaining nutritional quality may be as important as maintaining grain yield. The nutritional diversity present within wheat landraces offers potentially useful variation for breeding, but high baseline nutritional quality should not be equated with an ability to preserve that quality during drought or heat stress (Heidari et al., 2016; Hernandez-Espinosa et al., 2020).

Taken together, these findings broaden the concept of climate resilience from yield maintenance alone to the joint preservation of productivity and grain quality. Wheat landraces provide substantial variation in protein composition, mineral concentration, bioactive metabolites, and other quality-related traits, but the breeding value of this diversity will depend on how these characteristics interact with yield across contrasting environments. A landrace with exceptional nutritional composition but severe yield penalties may have limited direct agronomic value, whereas specific landrace alleles or trait combinations that improve nutritional quality without compromising yield stability could provide valuable breeding targets. Climate-resilient wheat improvement should therefore evaluate yield, nutritional composition, and stress performance as interconnected breeding objectives rather than selecting them independently.

## 7 Wheat landraces as genetic resources for climate-resilient wheat improvement

The adaptive traits described in the preceding sections make wheat landraces important sources of genetic variation for climate-resilient breeding. Their value lies not in an inherent superiority over modern cultivars, but in the retention of alleles and haplotypes that may be absent or poorly represented in elite breeding pools and that can contribute to adaptation under particular environmental conditions. Translating this diversity into improved cultivars nevertheless requires more than identifying stress-responsive traits: useful variation must be characterized, transferred into suitable breeding backgrounds, and validated for agronomic performance across target environments. Pre-breeding therefore provides a critical link between landrace diversity and cultivar development by enabling potentially beneficial variation to be incorporated while reducing undesirable agronomic characteristics and linkage drag (Lopes et al., 2015; Adhikari et al., 2022; Sukumaran et al., 2022; Shokat et al., 2023; Cheng et al., 2024).

### 7.1 Genetic diversity of wheat landraces: a reservoir of adaptive variation

The continuous selection of wheat under diverse agroecological conditions has generated extensive genetic diversity within landrace populations, enabling adaptation to contrasting environments over long evolutionary and cultivation histories. Unlike modern breeding programmes that have primarily emphasized high yield and uniformity, traditional farming systems often maintained genetically heterogeneous populations across locally contrasting environments. As a result, wheat landraces harbour genetic variants that may be absent or occur at lower frequencies in contemporary elite breeding material. This genetic richness is reflected in substantial variation for drought and heat tolerance, root system architecture, nutrient-use efficiency, disease resistance, phenological plasticity, and grain quality traits, many of which are relevant to adaptation and agronomic performance under stressful environments (Lopes et al., 2015; Adhikari et al., 2022; Shokat et al., 2023).

Recent genomic investigations have reinforced the value of wheat landraces as sources of genetic variation for wheat improvement. Comprehensive analyses of global germplasm collections identified alleles and haplotypes associated with agronomic and adaptive traits, including variants that are poorly represented in modern breeding material. For example (Cheng et al., 2024), demonstrated that systematic incorporation of landrace diversity substantially expands the available genetic repertoire for wheat improvement and enables the identification of haplotypes associated with complex agronomic and adaptive traits. Similarly, population genomic analyses of wheat landraces from the Southern Winter Wheat Region of China identified high levels of genetic diversity and distinct population structures reflecting long-term adaptation to local environments, further emphasizing their value for future breeding programmes (Liu et al., 2024).

The adaptive diversity preserved within wheat landraces extends beyond individual genes to encompass complex quantitative traits governed by multiple interacting loci. Such diversity provides breeders with opportunities to identify combinations of loci associated with stress-responsive traits, although their value ultimately depends on their effects within the recipient genetic background and target environment. The principal breeding challenge is therefore not simply to conserve this diversity, but to identify which components provide reproducible agronomic advantages and transfer them into breeding material without simultaneously introducing undesirable characteristics (Lopes et al., 2015; Adhikari et al., 2022; Cheng et al., 2024).

### 7.2 Pre-breeding strategies for the effective utilization of wheat landraces

Despite their exceptional genetic diversity and adaptive potential, wheat landraces may lack agronomic characteristics required for direct use in intensive modern production systems. Many landraces exhibit undesirable agronomic characteristics, including lower grain yield, lodging susceptibility, non-uniform maturity, excessive plant height, and inconsistent grain quality compared with elite cultivars. Consequently, the effective utilization of landraces requires pre-breeding, a strategic process that identifies valuable alleles from genetically diverse germplasm and transfers them into intermediate breeding populations while minimizing undesirable traits. This approach serves as a critical bridge between genetic resource conservation and practical cultivar development, allowing breeders to exploit adaptive while progressively recovering the agronomic performance required in elite breeding backgrounds (Lopes et al., 2015; Sukumaran et al., 2022; Shokat et al., 2023).

By incorporating genetically diverse landraces into breeding populations, pre-breeding programmes introduce alleles and haplotypes associated with drought and heat responses, disease resistance, nutrient-use efficiency, and performance under stressful environments. Selection within these populations can generate intermediate parental material combining selected landrace-derived traits with agronomic characteristics of elite germplasm. provement (Adhikari et al., 2022; Sukumaran et al., 2022; Cheng et al., 2024).

One of the major challenges in utilizing wheat landraces is the simultaneous transfer of favourable and unfavourable genomic regions, commonly referred to as linkage drag. Beneficial alleles associated with stress adaptation are frequently inherited together with undesirable traits, reducing breeding efficiency and delaying cultivar development. Pre-breeding programmes therefore employ repeated crossing, selection, and population development to progressively recover elite agronomic backgrounds while retaining adaptive genetic variation from landraces. Evidence of historical introgression among wheat lineages further illustrates how gene flow has contributed useful genetic variation to cultivated wheat, providing an evolutionary precedent for the deliberate incorporation of diverse germplasm into breeding populations (Cheng et al., 2019; Sertse et al., 2023).

Successful pre-breeding relies not only on identifying genetically diverse donor accessions but also on accurately characterizing adaptive phenotypes and selecting parental combinations that maximize useful variation. The increasing availability of well-characterized germplasm collections, together with improved phenotypic evaluation under contrasting environments, has enhanced the efficiency of identifying donor parents carrying target adaptive traitsfor climate-resilient breeding. The practical value of a landrace donor therefore depends not simply on the presence of a desirable allele or phenotype, but on whether that trait can be retained after transfer into an elite background without unacceptable penalties in yield, maturity, plant architecture, or grain quality and whether its advantage persists across the target environments of the breeding programme (Shokat et al., 2023; Cheng et al., 2024).

### 7.3 Integrating genetic resources into climate-resilient wheat improvement

The transition from landrace diversity to climate-resilient cultivar development requires a sequence of selection and validation steps. Candidate accessions must first be characterized for adaptive phenotypes and associated genetic variation, after which promising alleles or haplotypes can be introduced into suitable breeding backgrounds through pre-breeding. Their value must then be evaluated in the recipient background because the expression of complex traits such as drought tolerance, heat resilience, and yield stability can depend on genetic background and genotype × environment interactions. Multi-environment evaluation is therefore essential to determine whether a landrace-derived trait provides a reproducible agronomic advantage under the environmental conditions for which the resulting cultivar is intended (Lopes et al., 2015; Adhikari et al., 2022; Cheng et al., 2024; Mamrutha et al., 2026). This translation has been demonstrated using the Watkins wheat landrace collection, from which prioritized QTL were transferred into modern wheat breeding material, providing direct evidence that underutilized landrace diversity can be systematically mobilized for crop improvement (Cheng et al., 2024).

Importantly, breeding value should not be inferred from stress tolerance in the original landrace alone. Traits that enhance survival in a locally adapted population may carry penalties in flowering time, plant height, biomass allocation, grain quality, or yield when transferred into an elite background. Conversely, a donor showing only moderate stress tolerance may contain individual alleles or trait combinations that provide greater value when recombined with elite germplasm. Selection should therefore prioritize transferable adaptive components and their effects on yield stability rather than attempting to reproduce the complete landrace phenotype. This distinction is particularly important for complex traits controlled by multiple loci and strongly influenced by environmental conditions (Shokat et al., 2023; Sukumaran et al., 2022; Cheng et al., 2024). Direct introgression studies further demonstrate that exotic wheat diversity, including landrace-derived variation, can be incorporated into elite genetic backgrounds, while subsequent selection is required to identify derived lines combining useful donor variation with acceptable agronomic performance (Singh et al., 2021).

The efficient exploitation of this adaptive diversity is increasingly supported by high-throughput molecular technologies that enable precise identification of candidate alleles, haplotypes, regulatory pathways, and genomic regions associated with adaptive traits. For example, Deng et al. (2022) identified a stable adult-plant stripe-rust resistance QTL on chromosome 6AL in the Chinese wheat landrace Yibinzhuermai, illustrating how landrace variation can be resolved into specific genomic targets with potential breeding utility (Deng et al., 2022). The integration of genomics with other omics technologies can further connect genetic variation with molecular and physiological phenotypes, helping prioritize candidate traits for functional and field-level validation before their deployment in breeding programmes. These advances are discussed in the following section.

## 8 What modern science is revealing: omics insights into wheat landrace resilience

For many years, the value of wheat landraces was recognized primarily through field observations and agronomic performance. Farmers and researchers observed that certain landraces consistently performed well under drought, heat, poor soils, and other environmental constraints. The mechanisms underlying these phenotypes, however, cannot be resolved from field performance alone. Omics approaches now enable landrace variation to be examined across multiple biological layers, from genomic variants and stress-responsive transcription to protein abundance and metabolite accumulation. Importantly, evidence at these different levels does not carry equivalent breeding value: identifying a landrace-specific variant or molecular response establishes a candidate mechanism, whereas demonstrating its contribution to resilience requires functional validation and, ultimately, confirmation of agronomic effects under relevant field environments. This distinction provides the basis for evaluating how omics discoveries can move from molecular association to usable climate-resilience traits.

### 8.1 Genomics

Genomics has significantly expanded our understanding of the genetic diversity present within wheat landraces. High-throughput sequencing technologies have revealed that traditional wheat populations harbor substantial genetic variation including variants and haplotypes that are absent or poorly represented in modern breeding material (Liu et al., 2019; Cheng et al., 2024).

Genomic studies have identified substantial variation in genomic regions associated with environmental adaptation and agronomically important traits in wheat germplasm. Genome-wide association studies (GWAS) have been particularly useful for identifying loci associated with adaptive traits such as flowering time, root architecture, and stress-responsive physiological processes. Comparative analyses between landraces and modern cultivars revealed changes in genetic diversity and allele frequencies associated with domestication and subsequent breeding, highlighting genomic variation that may be underrepresented in contemporary breeding pools (Liu et al., 2019).

Large-scale genomic analysis of the Watkins wheat landrace collection provides a direct example of how genomic discovery can move beyond cataloguing diversity toward breeding application. Cheng et al. (2024) identified extensive landrace haplotype diversity and prioritized QTL for agronomically relevant traits, with selected landrace-derived variation subsequently transferred into modern wheat breeding material. This demonstrates an important strength of genomics: it can reduce large germplasm collections to defined genomic regions and candidate variation that can be tested within breeding populations rather than relying on the performance of entire landrace genomes (Cheng et al., 2024).

The principal limitation is that genomic association alone does not establish that a variant contributes to stress resilience; candidate loci require functional characterization and validation across relevant genetic backgrounds and environments before their breeding value can be established.

### 8.2 Transcriptomics

While genomics identifies genetic variation, transcriptomics provides insight into how plants respond to environmental stress through changes in gene expression. Transcriptomic comparisons have identified genotype-dependent differences in stress-responsive gene expression in wheat, providing a means of investigating regulatory responses associated with adaptation in landrace germplasm (Zou et al., 2015).

Studies investigating environmental stress responses in wheat have identified numerous stress-responsive genes involved in signaling pathways, osmotic adjustment, antioxidant regulation, hormone signaling, and cellular protection. Importantly, resilience is frequently associated not only with the presence of specific genes but also with the timing, magnitude, and coordination of their expression. Recent transcriptomic and co-expression network analyses of diverse wheat landraces have identified candidate regulatory genes associated withdrought and heat adaptation, highlighting the importance of complex regulatory networks in controlling stress responses (Barratt et al., 2023b, 2024).

Transcriptomic variation among wheat landraces suggests that different genotypes may recruit partly different regulatory responses under environmental stress, although whether these alternative expression patterns produce equivalent adaptive outcomes requires functional and field-level validation.These responses often involve transcription factor families such as DREB, NAC, WRKY, and bZIP, which regulate downstream genes associated with stress signaling, osmoprotection, and antioxidant defense. In addition, emerging evidence suggests that post-transcriptional regulatory mechanisms, including miRNA-mediated gene regulation, may contribute to adaptive responses in wheat landraces (Wu et al., 2025). Collectively, transcriptomic studies show that stress responses involve coordinated changes across multiple regulatory pathways rather than the action of individual genes alone. Their principal value for landrace research is therefore the identification of candidate regulatory networks that can subsequently be tested for functional effects and agronomic relevance.

### 8.3 Proteomics

Proteomics provides a direct assessment of the proteins responsible for cellular function and adaptation. Because proteins represent the active components of biological processes, proteomic analyses offer valuable insights into how wheat landraces respond to environmental challenges at the functional level. Research has shown that wheat responses to environmental stressoften involves coordinated changes in proteins associated with energy metabolism, photosynthesis, cellular protection, protein folding, and stress signaling (Moloi et al., 2024). Frequently identified stress-responsive proteins include heat-shock proteins, antioxidant enzymes, molecular chaperones, and proteins involved in osmotic adjustment and detoxification processes. Proteomic studies have further demonstrated that the abundance of these stress-responsive proteins can vary considerably among wheat genotypes exposed to similar environmental conditions (Skylas et al., 2001).

Comparative analyses have revealed differences in protein accumulation between Mediterranean wheat landraces and modern cultivars, demonstrating proteomic variation associated with contrasting germplasm backgrounds (Vita et al., 2016). Similarly, proteomic investigations of Afghan wheat landraces identified proteins associated with responses to Ca-rich alkaline soils, highlighting candidate mechanisms potentially contributing to performance under these edaphic conditions (Haque et al., 2025). Under drought conditions, proteins involved in oxidative stress defense, senescence regulation, and stem reserve remobilization have been associated with genotypic differences in drought response and grain-filling processes in wheat (Bazargani et al., 2011).

A potentially important contribution of proteomics is the identification of proteins that could serve as biomarkers of stress response. Their use in breeding, however, requires validation that protein abundance consistently predicts agronomic performance across genotypes, developmental stages, and target environments. Proteomics therefore provides an important functional layer between stress-responsive gene expression and plant phenotype, but direct proteomic comparisons linking landrace-specific protein responses with superior field performance remain a priority for future research.

### 8.4 Metabolomics

Metabolomics focuses on the small molecules produced during plant growth, development, and environmental responses. Because metabolites represent the final products of cellular processes, they provide a direct reflection of plant physiological status. Metabolomic studies have revealed substantial genotypic variation in wheat andraces in the accumulation of sugars, amino acids, organic acids, lipids, phenolic compounds, and other stress-related metabolites. These compounds contribute to osmotic adjustment, antioxidant defense, energy balance, membrane stability, and cellular protection, providing potential biochemical mechanisms through which wheat can respond to environmental stress (Cui et al., 2026).

Comparative metabolomic analyses have identified distinct metabolic profiles among wheat germplasm, including differences between traditional and modern materials, demonstrating substantial biochemical diversity associated with genotype and breeding history (Poudel et al., 2021). Similarly, metabolomic and lipidomic profiling of Tunisian durum wheat landraces distinct metabolic fingerprints and substantial diversity in lipid composition, demonstrating biochemical variation preserved within locally adapted germplasm (Lamine et al., 2022, 2023). Recent studies further suggest that wheat genotypes possess characteristic metabolic landscapes that may contribute to differences in stress adaptation and grain quality (Othibeng et al., 2025).

The breeding value of metabolomic variation depends on distinguishing metabolites that merely differentiate genotypes from those that consistently predict stress performance. A metabolic signature observed in a locally adapted landrace may reflect genetic background, developmental stage, or environmental history without necessarily contributing causally to resilience. Metabolomics becomes particularly informative when metabolite profiles are linked with physiological responses and yield performance under controlled and field stress conditions, allowing candidate biochemical markers to be evaluated for their stability and predictive value across environments.

### 8.5 Pangenomes and the discovery of hidden diversity

Traditional reference genomes capture only a portion of the genetic diversity present within a species. The development of wheat pangenomes has therefore represented a major advance in understanding landrace diversity. A pangenome includes both core genes shared by all individuals and variable genes that occur only within specific populations (Tiwari et al., 2024).

Pangenomic analyses have revealed that wheat landraces contain variable genomic regions, presence–absence variations, and structural variants that may not be represented in individual reference genomes. Some of this structural and presence–absence variation occurs within or near genomic regions associated with agronomically important and stress-responsive traits, creating candidate variation for further investigation. Consequently, pangenomes are uncovering previously hidden sources of diversity that may contribute to future crop improvement (Tiwari et al., 2024; Waqas et al., 2025).

The application of pangenomics has also highlighted the limitations of relying on a small number of elite cultivars as representatives of wheat diversity. By incorporating landraces into pangenome analyses, researchers can capture genomic variation that would remain undetected when relying on a single or limited number of reference genomes, expanding the pool of candidate variants available for gene discovery and breeding (Waqas et al., 2025). The principal challenge is therefore shifting from discovery to functional interpretation: structural or presence–absence variation identified through a pangenome should not be considered adaptive until its phenotypic effects are established and validated under relevant environmental conditions.

### 8.6 Integrating multi-omics for precision discovery of climate-resilience traits

The individual omics approaches discussed in the preceding sections have substantially improved our understanding of wheat landrace adaptation; however, each captures only one component of the complex biological processes underlying environmental resilience. Genomics identifies genetic variation, transcriptomics reveals stress-responsive gene regulation, proteomics characterizes functional protein abundance, metabolomics reflects physiological and biochemical responses, and pangenomics uncovers previously hidden genetic diversity. Integrating these complementary datasets can establish connections from genomic variation through transcriptional regulation, protein abundance, and metabolite accumulation to physiological and agronomic phenotypes. Such integration can therefore help distinguish candidate variants that merely differ among genotypes from molecular changes that are consistently associated with stress-responsive phenotypes (Mahmood et al., 2022; Kumar Saini and Kole, 2024).

The principal advantage of multi-omics is therefore not simply the accumulation of larger datasets, but the ability to test whether signals detected at different biological levels converge on the same adaptive phenotype. For example, a genomic variant associated with drought response becomes a stronger candidate when it is also linked to altered expression of the corresponding gene, changes in protein abundance or downstream metabolites, and a reproducible physiological response. Nevertheless, convergence across omics layers still represents evidence of association rather than definitive proof of adaptive function. Functional validation and evaluation of the resulting phenotype in relevant genetic backgrounds and field environments remain necessary before such candidates can be considered useful breeding targets. Combining pangenomics, transcriptomics, proteomics, metabolomics, phenotyping, and computational approaches can therefore provide a more rigorous framework for prioritizing landrace-derived variation for subsequent validation and breeding (Syeda, 2025; Yao et al., 2025).

Looking ahead, the increasing availability of high-throughput phenotyping, artificial intelligence, machine learning, and predictive analytical frameworks is expected to further enhance the interpretation of large-scale omics datasets. These emerging approaches can support the integration of multidimensional biological information and improve the prioritization of genotype–phenotype relationships and candidate donor germplasm for further evaluation. The practical value of these approaches will ultimately depend on whether molecular predictions translate into reproducible improvements in stress performance and yield stability under target environments (Yao et al., 2025; Tanin et al., 2026). For wheat landraces, the key research priority is therefore to move from omics-based discovery to a validated chain of evidence linking landrace-specific variation to molecular function, stress-responsive phenotype, and field-level agronomic performance.

## 9 From landrace to climate-smart wheat: translating resilience into breeding innovation

The increasing frequency of climate-related stresses has intensified the need for wheat cultivars capable of maintaining productivity under variable and challenging environmental conditions. Wheat landraces provide a valuable source of adaptive traits that can support this goal. However, identifying resilient landraces is only the first step. The greater challenge lies in transferring beneficial characteristics from traditional germplasm into modern breeding programs while maintaining agronomic performance and yield potential. Recent advances in genetics, genomics, and breeding technologies provide different but complementary routes for transferring validated landrace-derived variation into breeding populations, depending on the genetic architecture of the target trait and the breeding objective. The process of translating adaptive traits from wheat landraces into climate-smart cultivars through phenotypic and genomic characterization, trait validation, pre-breeding, selection, and precision improvement is summarized in **Figure 4**.

**Figure 4:**
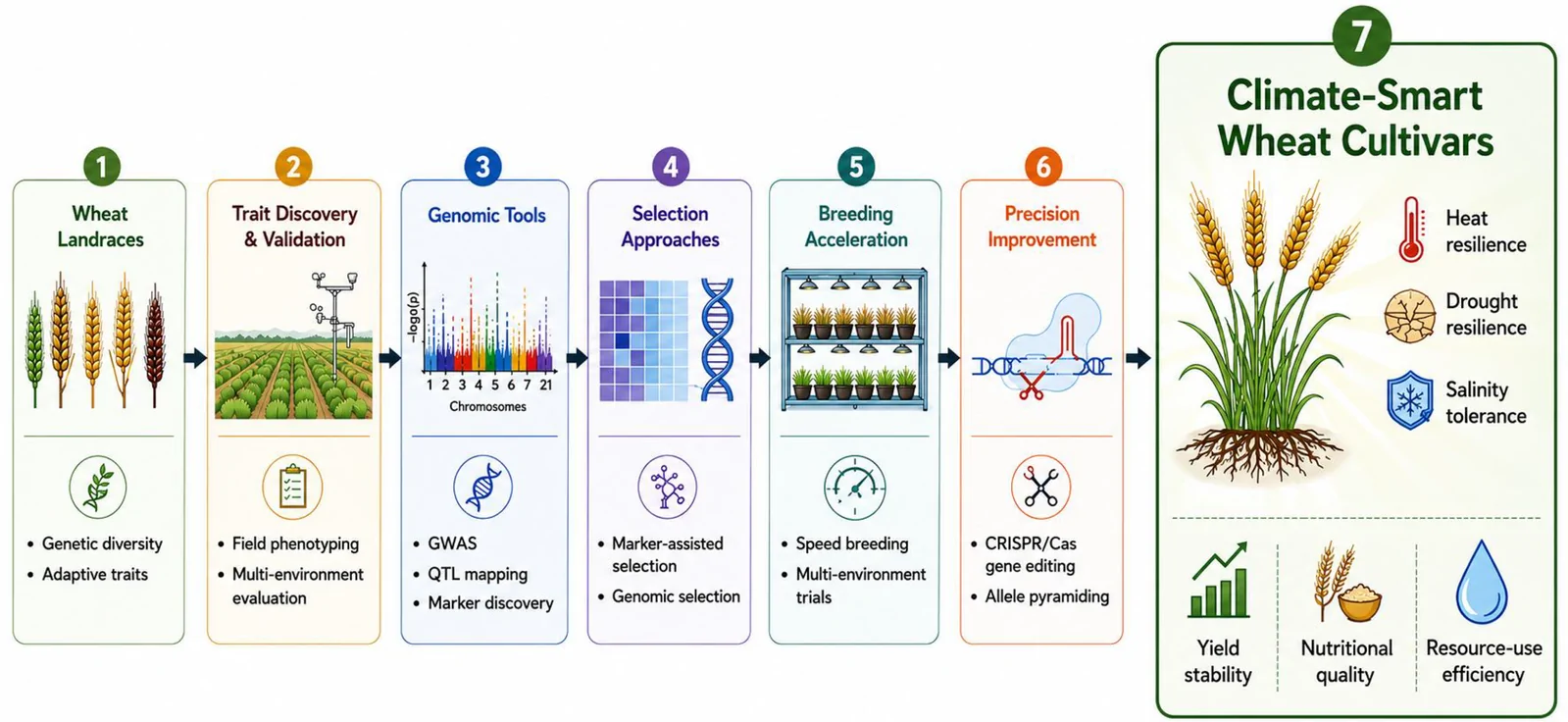
Conceptual pathway for translating wheat landrace diversity into climate-smart cultivars.

### 9.1 Trait discovery and characterization

Successful utilization of wheat landraces begins with the identification of traits associated with environmental resilience. Historically, trait discovery relied primarily on field observations and phenotypic evaluation. While these approaches remain fundamental, modern breeding increasingly integrates field assessments with physiological, genomic, transcriptomic, proteomic, and metabolomic analyses to better understand the mechanisms underlying adaptation (Cheng et al., 2024). Relevant breeding targets include yield stability under stress, efficient water and nutrient acquisition, adaptive phenology, maintenance of grain quality, and tolerance to drought, heat, salinity, and nutrient limitations. Many of these traits are complex and arise from interactions among morphological, physiological, and molecular processes (Adhikari et al., 2022). A major challenge in trait discovery is distinguishing traits that consistently contribute to adaptation across environments from those that are beneficial only under specific conditions. Multi-environment trials, combined with high-throughput phenotyping and omics-based analyses, can help determine whether candidate traits provide reproducible advantages across environments or represent environment-specific responses (Gharib et al., 2021). For breeding purposes, priority should therefore be given not simply to traits showing strong responses in the original landrace, but to those that are heritable, transferable to suitable breeding backgrounds, and associated with reproducible agronomic benefits under the target production environment.

### 9.2 Genome-wide association studies and marker discovery

Genome-wide association studies (GWAS) have become a key approach for identifying genomic regions associated with potentially useful traits within diverse wheat germplasm, including landraces. By exploiting historical recombination events within diverse germplasm collections, GWAS can enable the identification of genomic regions associated with adaptive traits (Liu et al., 2019). In wheat landraces, GWAS has facilitated the discovery of loci associated with agronomic, phenological, root, grain-quality, and stress-responsive traits.Associated loci can provide candidate markers for breeding, but their usefulness depends on validation of the marker–trait relationship in independent populations and relevant genetic backgrounds. However, many resilience-related traits are quantitative in nature and influenced by numerous loci with small effects. As a result, marker discovery alone is often insufficient to capture the full complexity of environmental adaptation For highly polygenic traits, GWAS-derived information can therefore complement rather than replace approaches such as genomic selection, which use genome-wide marker effects to predict breeding performance (Cheng et al., 2024).

### 9.3 Marker-assisted and genomic selection

The identification of molecular markers associated with adaptive traits can facilitate their application in wheat breeding programmes. Marker-assisted selection (MAS) uses DNA markers linked to desirable genes or quantitative trait loci (QTLs) to facilitate the efficient transfer of favorable alleles from landraces into elite germplasm. This approach is particularly effective for traits controlled by one or a small number of loci with sufficiently large and reproducible effects, and has been widely applied to the introgression of disease-resistance, quality-related, and other agronomically important alleles (Pandurangan et al., 2022; Song et al., 2023). However, many climate-resilience traits, including drought tolerance, heat adaptation, and yield stability, are governed by numerous loci with small individual effects. For such complex traits, genomic selection (GS) may be more appropriate, using genome-wide marker information to predict breeding values and identify superior candidates for advancement. MAS and GS should therefore be viewed as complementary rather than interchangeable strategies: MAS is particularly useful for transferring validated major-effect loci, whereas GS is better suited to capturing the cumulative effects of numerous loci underlying complex traits such as drought response and yield stability. For landrace-derived variation, the predictive value of either approach ultimately depends on the performance of the target allele or genomic background after introgression into elite material and across the environments in which the resulting breeding lines will be deployed.

### 9.4 Speed breeding and accelerated improvement

Traditional wheat breeding is often constrained by the time required to complete successive generations and evaluate breeding populations. Speed breeding has emerged as an approach for accelerating crop improvement by manipulating environmental conditions, including extended photoperiods, controlled temperatures, and optimized growth regimes, to shorten generation cycles (Cha et al., 2023). By enabling multiple generations to be advanced within a single year, speed breeding can reduce the time required to advance crosses, increase homozygosity, and generate breeding material for subsequent evaluation. For landrace pre-breeding, accelerated generation advancement could shorten the time required for repeated crossing and selection after candidate variation has been identified, thereby facilitating the recovery of agronomically suitable breeding backgrounds (Gao et al., 2025). When combined with marker-assisted selection, genomic selection, speed breeding can accelerate the advancement of selected material, while the identification and validation of resilience-associated traits remain dependent on phenotypic, genomic, and field evidence (Batista et al., 2024). Its principal contribution to landrace utilization is therefore temporal rather than biological: speed breeding can shorten the breeding cycle, but it cannot substitute for multi-environment evaluation of whether the transferred trait improves stress performance and yield stability.

### 9.5 Gene editing and precision breeding

Gene-editing technologies, particularly CRISPR-based systems, are expanding the possibilities for precision breeding in wheat. Unlike conventional introgression, which can transfer linked chromosomal regions together with a target allele, gene editing enables targeted modification of defined genes without requiring the transfer of large donor chromosomal segments (Li et al., 2021). The increasing availability of genomic and pangenomic information has improved the identification of candidate genes associated with stress adaptation, resource-use efficiency, plant architecture, and grain quality. This creates opportunities to evaluate and, where function has been sufficiently validated, modify candidate genes associated with adaptive or agronomically important traits identified in wheat landraces. Recent advances in CRISPR/Cas9, base editing, and prime editing further expand the range of genetic modifications that can be introduced with high precision (Li et al., 2021; Smedley et al., 2026).

One potential application of gene editing is the targeted improvement of valuable landrace germplasm without replacing the broader genetic background in which locally adapted variation is retained. Recent studies have demonstrated the successful introduction of semi-dwarf phenotypes into diverse wheat landrace backgrounds using CRISPR-based approaches, illustrating the potential for precision editing to modify specific agronomic characteristics while retaining much of the original landrace genetic background (Smedley et al., 2026). Gene editing is increasingly viewed as a complementary tool to conventional and genomic-assisted breeding rather than a replacement for these approaches. Its most appropriate role will depend on trait architecture: editing may be particularly useful where a defined gene has a validated functional effect, whereas polygenic traits may require genomic selection, recombination, and population-level improvement. Despite its considerable potential, several challenges remain. Many resilience traits are governed by complex regulatory networks involving multiple genes and gene–environment interactions, limiting the effectiveness of single-gene modifications. In addition, regulatory frameworks governing gene-edited crops vary considerably among countries, influencing the adoption, commercialization, and deployment of these technologies (Waites et al., 2025).

### 9.6 Toward climate-smart wheat cultivars

The ultimate objective of climate-resilient breeding is the development of cultivars capable of maintaining stable productivity, quality, and adaptation under increasingly variable environmental conditions. Wheat landraces can contribute valuable genetic variation for this effort because they harbor adaptive diversity accumulated through centuries of natural and farmer-driven selection (Babalola et al., 2025). The appropriate route from landrace diversity to cultivar development depends on the genetic architecture of the target trait: validated major-effect loci may be amenable to marker-assisted introgression or precision editing, whereas polygenic traits such as drought response and yield stability require population-level selection, genomic prediction, and evaluation across environments. Breeding technologies should therefore be selected according to the biological characteristics of the target trait rather than combined indiscriminately (Broccanello et al., 2023).

Ultimately, the success of landrace-based climate-resilient breeding should be measured not by the number of adaptive traits, molecular markers, or candidate genes identified, but by whether landrace-derived variation produces reproducible improvements in yield stability and other target traits after incorporation into elite genetic backgrounds. Multi-environment testing remains essential because the value of adaptive variation may change with stress intensity, developmental stage, soil conditions, and interactions among environmental constraints. The most useful landrace-derived traits will therefore be those that retain their advantages across the intended production environments without unacceptable penalties in yield potential, phenology, grain quality, or other agronomic characteristics. This evidence-based progression from diversity discovery to field-validated performance provides the basis for translating wheat landraces into practically useful resources for climate-smart wheat improvement.

## 10 Agronomic strategies supporting climate-resilient wheat production

The successful utilization of wheat landraces in climate-resilient agriculture depends not only on their genetic potential but also on the management practices used during cultivation. Arid and semi-arid environments are characterized by limited water availability, low soil organic matter, high evapotranspiration rates, and increasing climatic variability Sustainable cultivation practices that improve water availability, soil health, crop establishment, and resource-use efficiency may complement genetically determined adaptive traits and influence the field performance of landraces under arid and semi-arid conditions. The agronomic value of a landrace should therefore be considered as a genotype × management × environment outcome rather than as an intrinsic property of the genotype alone.

### 10.1 Water management strategies

Water scarcity remains the primary constraint to wheat production in many arid and semi-arid regions. Improving water-use efficiency is therefore essential for sustaining productivity under climate change. Deficit irrigation and supplemental irrigation strategies can be used to improve water productivity by targeting critical growth stages while reducing overall water consumption. In addition, precision irrigation technologies, including soil-moisture monitoring and sensor-based scheduling systems, provide opportunities to optimize water use and minimize losses. For wheat landraces, the effectiveness of these strategies is likely to depend on genotype-specific responses to the timing and intensity of water limitation, making genotype × irrigation interactions an important consideration when designing management strategies for arid environments (Saspugayeva et al., 2026). Landraces selected for drought-prone environments should therefore not be assumed to perform optimally under the same irrigation regime; field evaluation is needed to determine whether reduced or strategically timed irrigation complements their phenology, rooting characteristics, and yield response.

### 10.2 Conservation agriculture

Conservation agriculture is increasingly recognized as a management strategy that can contribute to resilience in dryland wheat production systems. The approach is based on three key principles: minimal soil disturbance, permanent soil cover, and diversified crop rotations. Reduced tillage can help preserve soil structure and moisture, residue retention can moderates soil temperature and reduces evaporation, and crop rotations can contribute to soil fertility while reducing pest and disease pressure. Together, these practices can improve soil-water conservation and buffer some of the effects of climatic variability, although their effectiveness depends on soil type, rainfall regime, residue availability, and management history.Whether conservation-agriculture practices provide additional benefits when combined specifically with locally adapted wheat landraces remains less well established and requires direct genotype × management comparisons under representative dryland conditions (Perera et al., 2026).

### 10.3 Seed priming as a climate adaptation tool

Seed priming is a simple and cost-effective technique that has gained considerable attention for improving crop establishment under stressful conditions. Controlled hydration treatments activate metabolic processes before sowing while preventing complete germination. Various approaches, including hydropriming, osmopriming, nutrient priming, and biopriming, can improve germination, seedling vigor, and early establishment under drought, salinity, and temperature stress (Ntshalintshali et al., 2025). Because successful crop establishment is particularly vulnerable to environmental stress in arid regions, seed priming may improve stand establishment when adverse conditions occur during germination and early seedling development (Abro et al., 2009). These early-stage benefits should not, however, be assumed to confer resilience throughout the crop cycle, and direct comparisons are needed to determine whether priming responses differ among wheat landraces and whether improved establishment translates into greater yield stability under field conditions.

### 10.4 Biochar for soil health and climate resilience

Biochar has emerged as a promising soil amendment for improving the sustainability and resilience of agricultural systems under water-limited conditions. Produced through the pyrolysis of biomass, biochar possesses a highly porous structure that can influence soil water-holding capacity, nutrient retention, cation exchange capacity, and microbial activity. These properties armay be beneficial in arid and semi-arid environments where low organic matter and poor soil fertility constrain wheat productivity. Biochar application can modify soil physical and chemical properties in ways that improve nutrient retention and soil-water availability, although crop responses depend strongly on biochar characteristics, application rate, soil type, and environmental conditions (Waheed et al., 2026).

Although biochar has been investigated in wheat production, direct evidence evaluating biochar × wheat-landrace interactions remains limited. A more relevant research priority is to test landrace × biochar interactions directly across contrasting soils and water regimes to determine whether particular landrace traits benefit from biochar-mediated changes in water and nutrient availability and whether these responses translate into improved yield stability.

### 10.5 Farmer seed systems and local knowledge

The long-term persistence of wheat landraces has depended largely on farmer-managed seed systems. Through seed selection, storage, exchange, and cultivation practices, farming communities have maintained locally adapted germplasm and preserved valuable genetic diversity. Farmer-managed seed systems also function as dynamic on-farm conservation networks, maintaining genetic diversity while allowing landrace populations to continue responding to farmer selection and local environmental conditions. This dynamic conservation complements *ex situ* germplasm collections by maintaining landraces within the farming systems in which selection, seed exchange, and environmental variation continue to shape population diversity.These systems remain particularly important in marginal agricultural regions where access to commercial seed is limited. In addition to conserving diversity, farmer seed networks facilitate the exchange of local knowledge related to crop management, adaptation strategies, and environmental stewardship. Strengthening these systems can help maintain landrace diversity and support resilient agricultural landscapes under changing climatic conditions (Bishaw et al., 2010; Chentoufi et al., 2014).

Water-efficient irrigation, conservation agriculture, seed enhancement technologies, biochar-mediated soil improvement, and farmer-managed seed systems address different components of production-system resilience, from water and soil management to crop establishment and maintenance of local germplasm diversity. Their contribution will nevertheless depend on genotype × management × environment interactions, emphasizing the need to evaluate landrace performance under the specific agronomic practices and environmental conditions in which the germplasm is intended to be deployed.The broader socio-economic and policy mechanisms required to support the conservation and adoption of wheat landraces are discussed in the following section (Jaradat, 2013).

## 11 Economic and policy perspectives

The future contribution of wheat landraces to climate-resilient agriculture depends not only on their biological and agronomic value but also on the economic and policy environments in which they are cultivated. Despite their adaptive potential, the continued use of landraces is often challenged by the widespread adoption of high-yielding cultivars, changing market demands, and the erosion of traditional farming systems. The long-term conservation and utilization of wheat landraces therefore depend on aligning farmer incentives and market opportunities with policy frameworks that support conservation, sustainable use, equitable access, and the integration of these genetic resources into climate-resilient agricultural systems.

### 11.1 Heritage grain markets and value creation

Growing consumer interest in sustainable foods, traditional farming systems, and nutritionally diverse products has created new opportunities for heritage grains. Ancient wheat species and traditional landraces can occupy differentiated or premium market niches based on their cultural identity, sensory characteristics, production systems, and perceived nutritional attributes. The revival of Einkorn in Türkiye illustrates how consumer interest in traditional foods can create new market opportunities for heritage wheat production.The development of niche markets linking small farmers with urban consumers illustrates how economic value creation can contribute directly to the conservation and continued cultivation of traditional wheat varieties (Atalan Helicke, 2024). Beyond direct market returns, wheat landraces also possess broader economic value through their contribution to genetic resource conservation, future breeding programs, cultural heritage, and agricultural biodiversity (Karabak and Kan, 2021). The conservation benefit of such markets nevertheless depends on whether economic returns reach the farmers maintaining the germplasm and provide sufficient incentive for its continued cultivation.

### 11.2 Farmer incentives and adoption

Farmers are the primary custodians of wheat landrace diversity. Their decisions regarding seed selection, cultivation practices, and crop replacement ultimately determine whether landraces are maintained or lost. Consequently, conservation efforts are more likely to support continued cultivation when they provide tangible benefits to farmers.Economic incentives may include premium prices, certification schemes, conservation payments, and support for local seed systems.Farmer decisions are also influenced by factors beyond direct financial returns. Studies have shown that motivations for maintaining crop landraces may also include cultural heritage, environmental stewardship, seed sovereignty, and the preservation of locally adapted genetic resources (Ortman et al., 2023). In addition, extension services, technical support, and improved market access can support the continued cultivation of traditional varieties. Policies that strengthen the role of farmers in conserving agricultural biodiversity will be most relevant when incentives address the practical reasons for landrace replacement, including yield differences, market access, seed availability, and production risk. The practical challenge is therefore to make conservation economically compatible with farmers’ production objectives rather than treating continued landrace cultivation as a conservation responsibility borne primarily by farming communities.

### 11.3 Conservation policies and genetic resource management

National and international policies play a central role in the conservation and utilization of wheat genetic resources. Conservation efforts have traditionally focused on *ex situ* collections maintained in national and international genebanks, which provide an essential safeguard against genetic erosion and preserve valuable genetic diversity for future breeding programs (Adhikari et al., 2022). Studies of wheat landrace collections have revealed substantial genetic variation both within and among accessions, highlighting the importance of systematic conservation and genetic resource management (Dreisigacker et al., 2005). Conservation frameworks increasingly recognize the importance of complementing *ex situ* collections with on-farm and *in situ* conservation, where landraces continue to evolve under local environmental and management conditions (Guzzon et al., 2022). Effective conservation strategies therefore require coordinated support for germplasm characterization, documentation, appropriate mechanisms for seed access and seed exchange, farmer participation, and long-term monitoring of genetic diversity. As climate change increases the demand for adaptive genetic resources, should support not only the preservation of wheat landraces but also their characterization, evaluation, and responsible utilization, so that conserved diversity remains accessible for research, breeding, and cultivation (Guzzon et al., 2022).

Neither *ex situ* nor on-farm conservation alone is sufficient to safeguard wheat landrace diversity. *Ex situ* collections preserve genetic material but cannot maintain the continuous evolutionary processes driven by farmer selection and changing environmental conditions. Conversely, on-farm conservation allows populations to remain subject to farmer selection and changing production environments but may expose landraces to genetic erosion or replacement by modern cultivars. Consequently, complementary conservation strategies linking genebank preservation with farmer-managed on-farm conservation can protect stored genetic diversity while maintaining opportunities for continued selection and use under changing agricultural environments. A central policy challenge is therefore not simply to maximize the number of conserved accessions, but to connect conservation with characterization, evaluation, farmer access, and breeding use so that potentially valuable landrace diversity can move from preservation to practical deployment.

### 11.4 Access, benefit sharing, and farmers’ rights

The utilization of wheat landraces in research and breeding raises important questions regarding access, ownership, and the equitable distribution of benefits. Many landraces have been maintained by farming communities over generations, and their use in crop improvement programs often relies on genetic resources and traditional knowledge developed through long-term farmer stewardship. International frameworks such as the International Treaty on Plant Genetic Resources for Food and Agriculture provide mechanisms for facilitated access and benefit sharing for specified plant genetic resources while also recognizing Farmers’ Rights associated with their conservation and sustainable use (Adebola, 2019). Fair and equitable benefit-sharing mechanisms are increasingly recognized as essential components of sustainable genetic resource management. Recognition of farmer contributions, transparent access arrangements, and support for local communities can help ensure that the benefits derived from landrace utilization are shared more broadly while encouraging continued participation in conservation efforts (Tsioumani, 2020). The relationship between Farmers’ Rights, access and benefit-sharing arrangements, and intellectual-property frameworks also becomes increasingly important as landrace-derived genetic resources are incorporated into commercial breeding and advanced breeding technologies (Nhemachena et al., 2019). Supportive economic and policy frameworks are therefore needed to ensure that wheat landraces can be conserved while also remaining accessible for responsible research, breeding, and cultivation, with appropriate recognition of the farming communities that have contributed to their continued maintenance. The practical value of wheat landrace diversity therefore depends on more than identifying adaptive traits or conserving germplasm. Market incentives must make continued cultivation viable for farmers, conservation policies must maintain diversity while enabling its evaluation and use, and access and benefit-sharing frameworks must support responsible utilization of landrace-derived resources. Aligning these economic and policy mechanisms with breeding and agronomic strategies is essential for converting conserved landrace diversity into a resource that can contribute to climate-resilient wheat production.

## 12 Research gaps and future directions

Despite growing interest in wheat landraces as sources of climate resilience, major gaps remain between the identification of potentially adaptive variation and its validation and utilization in breeding and field production.Closing these gaps requires research that moves beyond descriptive characterization toward comparative, mechanistic, and field-validated evaluation of landrace-derived traits.

### 12.1 Understanding climate resilience under combined environmental stresses

Most studies continue to evaluate drought, heat, or salinity independently, even though these stresses frequently occur sequentially or simultaneously under field conditions. Future research should prioritize multi-stress experiments reproducereproduce realistic stress combinations, sequences, intensities, and developmental timing while explicitly quantifying genotype × environment interactions.that better reflectstress combinations, sequences, intensities, developmental timing while explicitly quantifying×. Integrating genomics, transcriptomics, proteomics, metabolomics, and field phenotyping could help determine whether molecular responses identified under individual stresses remain relevant when stresses occur in combination and whether these responses predict agronomic performance A key priority is therefore to identify landrace-derived traits that retain their value across multiple stress scenarios rather than conferring an advantage only under a single controlled stress condition (Babalola et al., 2025). Direct comparisons among landraces, modern cultivars, and derived breeding populations under the same combined-stress environments are particularly needed to determine whether putative landrace advantages are heritable, transferable, and agronomically meaningful.

### 12.2 Uncovering belowground mechanisms of adaptation

Although root systems and associated microbiomes play fundamental roles in resource acquisition and stress responses, their contribution to environmental resilience remains comparatively poorly characterized in wheat landraces. Future investigations should combine characterization of root architecture and plasticity with rhizosphere and microbiome analyses under controlled and field conditions, particularly in arid and semi-arid environments. Root traits such as root depth, growth angle, branching patterns, and root system distribution can influence soil exploration and water acquisition, but their adaptive value is likely to depend on soil profile, water distribution, developmental stage, and the target drought environment (Tao et al., 2024). Advances in root phenotyping and microbiome analysis should therefore be coupled with measurements of water acquisition, nutrient uptake, physiological performance, and grain yield to determine which belowground characteristics provide reproducible agronomic benefits. Particularly important are common-environment comparisons among landraces and modern cultivars, which can help distinguish genetically associated differences in root and microbiome traits from responses caused primarily by soil, location, or management history.

### 12.3 Artificial intelligence and digital phenotyping

Emerging AI-driven approaches, including knowledge graphs, deep learning, and integrative data analyticsprovide new opportunities to integrate genomic, phenotypic, environmental, and management data and identify complex relationships relevant to breeding decisions (Xie et al., 2026). Their effectiveness depends on sufficiently large, standardized, and well-characterized datasets representing diverse wheat landraces, environments, developmental stages, and management conditions.Future research should prioritize interoperable datasets that connect high-throughput phenotyping with environmental metadata, management information, and multi-omics profiles, together with predictive models that can be independently validated across populations and environments.Such integration could improve the prediction of genotype × environment responses and help prioritize landrace-derived traits or accessions for experimental validation, but predictive performance must be demonstrated on germplasm and environments not represented in model training (Xie et al., 2026). A particularly important research need is the development of landrace-representative training datasets, because models dominated by modern breeding germplasm may not adequately capture the genetic and phenotypic diversity present in traditional wheat populations.

### 12.4 Participatory and evolutionary breeding

Future breeding efforts should strengthen farmer participation and on-farm evaluation so that selection incorporates local production constraints, management conditions, and farmer-defined priorities alongside conventional breeding criteria. Farmers often possess valuable knowledge regarding environmental constraints, management practices, and preferred crop characteristics that can complement conventional breeding programs (Adhikari et al., 2022).

Evolutionary breeding approaches, in which genetically diverse populations continue to adapt under field conditions over successive generations of natural and farmer-mediated selection, allowing population composition to change in response to local environmental and management conditions.By maintaining genetic diversity within breeding populations, these approaches may provide greater scope for selection under variable environments, although changes in population composition do not necessarily guarantee improvements in yield, stability, or other target traits (Babalola et al., 2025). Participatory and evolutionary approaches therefore provide complementary rather than equivalent pathways: participatory breeding can improve the relevance of selection to farmer and target-environment priorities, whereas evolutionary approaches can maintain heterogeneous populations under continuing selection. For wheat landraces, future studies should compare these approaches with conventional breeding under the same target environments and quantify whether they improve yield stability, stress performance, farmer acceptance, and retention of useful genetic diversity over successive generations.

## 13 Conclusions

Climate change is imposing increasing challenges on global wheat production, particularly in arid and semi-arid regions where increasing temperatures, recurrent droughts, soil degradation, and environmental variability threaten crop productivity and food security. Addressing these challenges requires the development of wheat cultivars capable of maintaining stable performance under increasingly unpredictable environmental conditions. In this context, wheat landraces represent one of the most valuable yet underutilized reservoirs of genetic and phenotypic variation with potential value for climate-resilient wheat improvement. Shaped through centuries of natural selection and farmer-led selection under diverse agroecological conditions, these traditional populations contain variation in phenology, root characteristics, physiological and biochemical stress responses, and grain-quality traits that provides candidate diversity for adaptation to drought, heat, and other environmental constraints.

This review highlights that the resilience of wheat can arise through interacting genetic, molecular, physiological, biochemical, and belowground processes rather than through a single adaptive mechanism.Adaptive strategies such as stress escape, avoidance, tolerance, and recovery represent distinct but potentially interacting strategies whose benefits and trade-offs depend on stress type, timing, intensity, developmental stage, and target environment; root architecture and rhizosphere interactions add further context-dependent components of this response. Advances in genomics, transcriptomics, proteomics, metabolomics, and pangenomics are expanding the discovery of candidate alleles, regulatory responses, proteins, metabolites, and structural variation associated with stress-responsive phenotypes, but their breeding value depends on functional and agronomic validation. Translation into improved cultivars therefore requires an evidence chain linking landrace-derived variation to molecular or physiological function, reproducible stress performance, transferability into suitable breeding backgrounds, and ultimately yield stability under target field environments.Realizing the potential of wheat landraces also requires a attention beyond molecular discovery and breeding innovation. Sustainable agronomic management, farmer participation, effective conservation of genetic resources, supportive policy frameworks, equitable benefit-sharing mechanisms influence whether potentially useful landrace diversity can move from conservation and characterization into breeding and agricultural use. Future research should prioritize realistic combined- and sequential-stress experiments, common-environment comparisons of landraces and modern cultivars, root–rhizosphere phenotyping, representative multi-omics datasets, and multi-environment field validation to determine which landrace-derived traits provide reproducible agronomic advantages.

As global agriculture confronts an increasingly uncertain climatic future, wheat landraces should no longer be regarded as relics of traditional agriculture but as dynamic genetic resources for future crop improvement. Their conservationrigorous characterization, validation, and strategic utilization can contribute to the development of wheat cultivars better suited to increasingly variable and resource-limited production environments.The challenge now is to move beyond recognizing landraces as reservoirs of diversity and establish, through comparative and field-validated evidence, which components of that diversity can be translated into measurable gains in climate resilience, yield stability, and sustainable wheat production.
